# DNA copy number evolution in *Drosophila* cell lines

**DOI:** 10.1186/gb-2014-15-8-r70

**Published:** 2014-08-28

**Authors:** Hangnoh Lee, C Joel McManus, Dong-Yeon Cho, Matthew Eaton, Fioranna Renda, Maria Patrizia Somma, Lucy Cherbas, Gemma May, Sara Powell, Dayu Zhang, Lijun Zhan, Alissa Resch, Justen Andrews, Susan E Celniker, Peter Cherbas, Teresa M Przytycka, Maurizio Gatti, Brian Oliver, Brenton Graveley, David MacAlpine

**Affiliations:** National Institute of Diabetes, Digestive, and Kidney Diseases, National Institutes of Health, 50 South Drive, Bethesda, MD 20892 USA; Department of Genetics and Developmental Biology, Institute for Systems Genomics, University of Connecticut Health Center, 400 Farmington Avenue, Farmington, CT 06030 USA; Computational Biology Branch, National Center for Biotechnology Information, National Library of Medicine, National Institutes of Health, 8600 Rockville Pike, Bethesda, MD 20892 USA; Department of Pharmacology and Cancer Biology, Duke University Medical Center, Levine Science Research Center, 308 Research Drive, Durham, NC 27708 USA; Istituto di Biologia e Patologia Molecolari (IBPM) del CNR and Dipartimento di Biologia e Biotecnologie, Sapienza, Università di Roma, 5 Aldo Moro Piazzale, Rome, 00185 Italy; Department of Biology, Indiana University, 1001 East 3rd Street, Bloomington, IN 47405 USA; Department of Genome Dynamics, Lawrence Berkeley National Laboratory, 1 Cyclotron Road, Berkeley, CA 94720 USA; Department of Biological Sciences, Carnegie Mellon University, 4400 Fifth Avenue, Pittsburgh, PA 15213 USA; School of Agricultural and Food Science, Zhejiang A&F University, 88 Huan Cheng Bei Road, Lin’an, Zhejiang 311300 China

## Abstract

**Background:**

Structural rearrangements of the genome resulting in genic imbalance due to copy number change are often deleterious at the organismal level, but are common in immortalized cell lines and tumors, where they may be an advantage to cells. In order to explore the biological consequences of copy number changes in the *Drosophila* genome, we resequenced the genomes of 19 tissue-culture cell lines and generated RNA-Seq profiles.

**Results:**

Our work revealed dramatic duplications and deletions in all cell lines. We found three lines of evidence indicating that copy number changes were due to selection during tissue culture. First, we found that copy numbers correlated to maintain stoichiometric balance in protein complexes and biochemical pathways, consistent with the gene balance hypothesis. Second, while most copy number changes were cell line-specific, we identified some copy number changes shared by many of the independent cell lines. These included dramatic recurrence of increased copy number of the PDGF/VEGF receptor, which is also over-expressed in many cancer cells, and of *bantam*, an anti-apoptosis miRNA. Third, even when copy number changes seemed distinct between lines, there was strong evidence that they supported a common phenotypic outcome. For example, we found that proto-oncogenes were over-represented in one cell line (*S2-DRSC*), whereas tumor suppressor genes were under-represented in another (*Kc167*).

**Conclusion:**

Our study illustrates how genome structure changes may contribute to selection of cell lines *in vitro*. This has implications for other cell-level natural selection progressions, including tumorigenesis.

**Electronic supplementary material:**

The online version of this article (doi:10.1186/gb-2014-15-8-r70) contains supplementary material, which is available to authorized users.

## Background

### Copy number

While genes do generally come in pairs, there are a number of situations where gene copy number deviates from fully diploid [1]. Some of these deviations are normal, such as occurs in the case of sex chromosomes [2] and amplification in terminally differentiated cells [3, 4]. Polyploidy is also a whole chromosome-level copy number change that alters phenotypes in organisms such as plants and honey bees with distinct ploidy-specific morphs [5, 6]. In most situations, copy number changes are abnormal and deleterious, and vary in extent from full chromosomes, to chromosome segments, to focal regions altering the copy number of single genes. Karyotypically obvious copy number changes are usually referred to as aneuploidy. Submicroscopic copy number changes of limited extent along a chromosome are often referred to as copy number variants. Recent advancement of genome-wide techniques has made the detection of copy number much easier, and the extent of copy number variants in populations is extensive [7, 8].

Mechanisms responsible for different copy number classes vary. The major cause of whole chromosomal copy number change is mis-segregation at mitosis or meiosis, due to non-disjunction, checkpoint defects, cohesion defects, merotelic attachment of microtubules to kinetochores, multipolar mitotic spindles, or recombination or repair events generating dicentric and acentric chromosomes [9, 10]. Segmental copy number changes result from rearrangements due to repair events, unbalanced segregation of translocations to generate duplication and deletion pairs and recombination at tandem duplications [11–13]. These copy number events can be extensive, resulting in large copy number blocks, but are particularly informative when only a few loci are affected. Such small extent copy number changes are often found associated with repeats that promote non-allelic homologous recombination, while recombination mediated by 2 to 15 bp segments of microhomology can generate more sporadic changes in copy number [13]. While one can debate whether 2 bp is truly homologous, in both cases regions of extended or limited homology facilitate rearrangements during DNA repair.

At the organismal level, changes in copy number are often associated with a range of abnormalities, including death, developmental defects or delay, psychiatric disorders, spontaneous abortions, and cancers [11, 14]. Some copy number changes are the ‘drivers’ with phenotypic consequences, while others are neutral or nearly neutral ‘passengers’ [15–18]. When copy number changes are extensive (for example, monosomic chromosomes) multiple drivers are probable, but when copy number changes are limited in extent, and recurrent, it may be possible to deduce the identity of the driver genes associated with a particular phenotype. Additionally, in both *Drosophila* and humans, extensive copy number change results in death during development [19, 20]. In *Drosophila* this is unlikely due to specific drivers, but rather the additive effect of multiple copy number changes [21].

The effect of copy number change on fitness is context-dependent. For example, in crop plants polyploids often produce larger fruits or flowers [22]. Unbalanced copy number changes result in more severe phenotypic changes than polyploidy, underscoring the importance of gene dosage balance, rather than absolute copy number [23]. In micro-organisms such as *Candida albicans* altered copy number of genes is believed to mediate antibiotic resistance [24]. Similarly, in tumor cells copy number changes resulting in favorable copy number configurations of drivers are associated with resistance to chemotherapy [25]. Indeed, direct experimental evidence shows that tumor cells gain advantages from chromosomal and segmental copy number changes, as a knockout of mitotic checkpoint components in mice increases both copy number deviations and spontaneous or carcinogen-induced tumorigenesis [9]. This link between copy number and cancer cell fitness is supported by high-throughput profiling of 8,000 cancer genomes, where pan-lineage alterations have been linked to kinases and cell cycle regulators [18]. These studies suggest that copy number changes can increase cellular fitness.

### *Drosophila*chromosomes

Euploid *Drosophila melanogaster* cells are diploid, with three pairs of autosomes and one pair of sex chromosomes, with females having two Xs and males having a single X and a Y chromosome. The number of X chromosomes determines sex [26], and the X chromosome is dosage compensated by association with the male-specific lethal (MSL) complex [27, 28]. The Y chromosome is required for male fertility but not viability and XX females bearing a Y are viable and fertile [29]. The small fourth chromosome is often monosomic, and is compensated by Painting of fourth (POF) [30]. To understand the biological effects of copy numbers, we studied genome structures of *D. melanogaster* tissue-culture cells. As previously demonstrated by resequencing *S2* cells [31], we found extensive copy number changes in these lines. Our data strongly support the idea that copy number change alters pathway function to select for increased growth, and that coherent copy number changes in genes encoding members of protein-protein complexes correct for imbalances to maintain complex function. Similarly, we suggest that selection against deleterious copy number effects result in regions where copy number changes are rare.

## Results

To determine copy number genome-wide, we performed next generation DNA sequencing (DNA-Seq) on naked DNA harvested from 19 modENCODE cell lines [32–41] and control DNA from adult females (Table 1). We then mapped the sequence reads to release 5 of the *D. melanogaster* reference genome to identify the relative copy number of each gene. In two cases, we resequenced libraries made from independent cultures, grown in different labs (*S2-DRSC* and *Cl.8*) to assay copy number stability, and found excellent agreement. For the *Cl.8* line, we found that the overall genome copy number structure was 99.6% identical. For the highly rearranged *S2-DRSC* line, we observed 87.2% copy number agreement between two independent cultures, suggesting that even these highly aberrant copy number states are relatively stable. Below, we describe the structure of these genomes in order of degree of copy number change.

**Table 1:** **modENCODE cell lines used in this study**

Official name	Short name	Tissue origin	Origin genotype	Clonal status	Reference
1182-4H	1182-4H	Embryo	mh	Not cloned; grown sparingly since establishment	[32]
ML-DmBG3-c2	BG3-c2	L3 CNS	y^1^ v^1^ f^1^ mal^F1^	Cloned; grown sparingly since cloning	[33]
CME W1 Cl.8+	Cl.8	L3 wing disc	Oregon R	Cloned; grown moderately since cloning	[34]
ML-DmD16-c3	D16-c3	L3 wing disc	y^1^ v^1^ f^1^ mal^F1^	Cloned; grown sparingly since cloning	[35]
ML-DmD17-c3	D17-c3	L3 haltere disc	y^1^ v^1^ f^1^ mal^F1^	Cloned; grown sparingly since cloning	[35]
ML-DmD20-c2	D20-c2	L3 antennal disc	y^1^ v^1^ f^1^ mal^F1^	Cloned; grown sparingly since cloning	[35]
ML-DmD20-c5	D20-c5	L3 antennal disc	y^1^ v^1^ f^1^ mal^F1^	Cloned; grown sparingly since cloning	[35]
ML-DmD4-c1	D4-c1	L3 mixed discs	y^1^ v^1^ f^1^ mal^F1^	Cloned; grown sparingly since cloning	[35]
ML-DmD8	D8	L3 wing disc	y^1^ v^1^ f^1^ mal^F1^	Not cloned; grown sparingly since establishment	[35]
ML-DmD9	D9	L3 wing disc	y^1^ v^1^ f^1^ mal^F1^	Not cloned; grown sparingly since establishment	[35]
Kc167	Kc167	Embryo	e/se	Cloned; grown very extensively since cloning	[36, 37]
CME L1	L1	L3 leg disc	Oregon R	Not cloned; grown sparingly since establishment	[34]
mbn2	mbn2	L3 hemocytes	l(2)mbn	Not cloned; grown moderately since establishment	[38]
S1	S1	Embryo	Oregon R	Not cloned; grown moderately since establishment	[39]
S2-DRSC	S2-DRSC	Embryo	Oregon R	Not cloned; grown very extensively since establishment	[39]
S2R+	S2R+	Embryo	Oregon R	Not cloned; frozen for >25 years, then grown sparingly	[39, 40]
S3	S3	Embryo	Oregon R	Not cloned; grown moderately since establishment	[39]
Sg4	Sg4	Embryo	Oregon R	Cloned; grown moderately since cloning	[39]
W2	W2	Wing disc	Oregon R	Not cloned; grown sparingly since establishment	[34]

### Ploidy of cell lines

**Figure 1:**
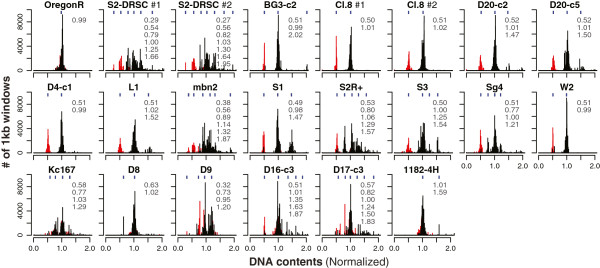
**Cell line ploidy by DNA-Seq.** Histograms of normalized DNA read density of 1 kb windows. Red, reads from X chromosomes; black, reads from autosomes; blue, centers of individual peak clusters; gray, peak cluster ratios. #1 and #2 indicate the results from two independent sets of DNA-Seq from different labs.

We first determined basal genome ploidy status from ratiometric DNA-Seq data. We took advantage of the extensive copy number deviations in the cell lines to make this determination. In our DNA-Seq analysis of the cell lines, we set the mean peak of DNA-Seq read count density at ‘1’ to reflect the relative nature of the measurements and plotted X-chromosome and autosomal DNA-Seq densities separately (Figure 1). DNA density ratios from different copy number segments can be represented as fractions with a common denominator and the smallest such denominator indicates the minimum ploidy. One good illustration was the *S1* cell line. We observed a DNA-density peak at 1.47 from DNA-Seq of *S1* cells, suggesting that a segmental duplication of autosomal DNA occurred in this line (approximately 50% increase) on a baseline diploid karyotype, since there was no DNA block with intermediate DNA content between approximately 1.5 and 1. Another example is *Kc167* cells, which had at least four levels of relative read-count ratios centered on 0.58, 0.77, 1.03 and 1.29. This distribution of DNA densities was consistent with tetraploidy. In the majority of cases, this simple analysis yielded a clear ploidy estimate. We scored *BG3-c2*, *Cl.8*, *D20-c2*, *D20-c5*, *D4-c1*, *L1*, *S1*, *W2*, and *D8* cell lines as minimally diploid, and *S2-DRSC*, *S2R+*, *S3*, *Sg4*, *Kc167*, *D16-c3*, and *D17-c3* cell lines as minimally tetraploid. Our results for *D9* and *mbn2* cell line ploidy were inconclusive, due to the presence of multiple regions of relative read densities that were not ratios of whole numbers.

**Figure 2:**
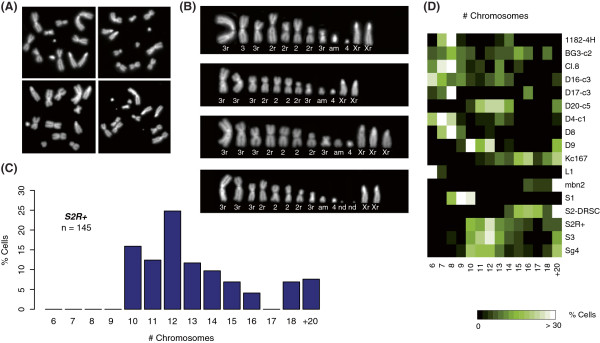
**Karyotypes. (A,B)** Metaphase spread figures of *S2R +* cells **(A)** and as aligned in karyograms **(B)**. Either wild-type, or close to wild-type chromosome 2 s and 3 s are designated with ‘2’ and ‘3’. If rearrangements were found on them, such as deletions, inversion or translocations, they are marked with ‘r’ (2r and 3r). Small chromosomes that carried euchromatic material appended to a centromeric region that was likely to derive from a large autosome are labeled as ‘am’. Chromosomes whose origin could not be determined are labeled ‘nd’. **(C)** Chromosome numbers in metaphases from 145 *S2R +* cells. **(D)** A heatmap summarizing chromosome numbers. Metaphase spreads for all the cell lines are provided in Additional file 1.

Ratiometric DNA-Seq data allowed us to determine minimal ploidy, but not absolute ploidy. Therefore, we also examined mitotic spreads (Figure 2; Additional files 1 and 2) to make ploidy determinations. In contrast with relativistic DNA-Seq measurements, mitotic chromosomes can be counted directly to determine chromosome number, although it is not always possible to determine exact chromosome identity due to rearrangements. We observed that *S1*, *Kc167*, *S2-DRSC*, *S2R+*, *S3* and *D20-c5* were tetraploids. *BG3-c2* and *1182-4H* cells were diploid. The DNA-Seq read ratio patterns for *D20-c5* suggested minimal diploidy, not tetraploidy, which may be due to a whole genome duplication following establishment of a relative copy number profile as detected by DNA-Seq.

Interestingly, the karyotypes of individual cells varied in all lines (Figure 2; Additional file 1). *Prima facie*, the variable numbers of chromosomes in the cells is in disagreement with the consistency of the DNA-Seq calls. For example, DNA-Seq results indicated tetraploidy for *D17-c3* cells, yet the karyogram showed a mixed state with diploid and tetraploid cells. Despite these heterogeneous ploidies, the DNA-Seq values for independent cultures (separated by an unknown, but presumed large number of passages) showed good agreement. These data suggest that even if the cell-to-cell karyotypes differ, the distribution of karyotypes is stable in the population of cells from a given line.

### Chromosomal gains and losses in cell lines

We identified frequent numeric aberrations of the X, Y, and fourth chromosomes. X chromosome karyotype is a natural copy number deviation that determines sex in *Drosophila*. Sexual identity is fixed early in development by *Sex-lethal* (*Sxl*) autoregulation [42], so deviations in the X chromosome to autosome (X:A) ratio that may have occurred during culture are not expected to result in a change in sex. Therefore, we used DNA-Seq-derived copy number and then expression of sex determination genes in expression profiling experiments (RNA-Seq) to deduce if the X chromosome copy was due to the sex of the animal from which the line was derived, or if the copy number change was secondary during culture.

In control females (Figure 1), there was a single peak of DNA read density centered on approximately 1 regardless of whether the reads mapped to the X chromosome or to autosomes. In the cell lines there were clear cases of X:A = 1 (that is, female), X:A = 0.5 (that is, male), and some intermediate values. DNA-Seq results for the *S2-DRSC*, *BG3-c2*, *Cl.8*, *D20-c2*, *D20-c5*, *D4-c1*, *L1*, *mbn2*, *S1*, *S3*, *Sg4* and *W2* lines showed under-representation of reads mapping to the X chromosome (X:A <0.75), suggesting that they are male, or female cells that have lost X chromosome sequence. Similarly, by these criteria *Kc167*, *D8*, *D9*, *D16-c3* and *D17-c3* cells appear to be female (X:A >0.75), but might also be male with extensive X chromosome duplications. Cytological analysis confirmed these findings (Additional file 1).

To determine sexual identity we analyzed the expression of sex-determination genes and isoforms from RNA-Seq data compared to those from 100 different lines of sexed *D. melanogaster* adults (Table 2). In *Drosophila*, the MSL complex (MSL-1, MSL-2, MSL-3, MLE proteins, and *RoX1* and *RoX2* non-coding RNAs) localizes to the X chromosome and hyper-activates gene expression to balance transcription levels to that of autosomes [43]. The alternative splicing of *Sxl* pre-mRNAs controls SXL protein production, which in turn regulates MSL formation by modulating *msl-2* splicing and protein levels. *Sxl* also regulates sex differentiation via the splicing of *transformer* (*tra*) pre-mRNA [44, 45]. Except for *D9* cells, we observed that the two RNA components of the male-specific MSL complex (*roX1* and *roX2*) genes were expressed at female levels in the cell lines with X:A >0.75 (*Kc167*, *1182-4H*, *D8*, *D16-c3*, and *D17-c3*), suggesting that observed DNA-Seq copy number values were due to the female identity of the cells used to establish these cultures. Similarly, cell lines that had an X:A <0.75 (*D4-c1*, *BG3-c2*, *Cl.8*, *D20-c5*, *L1*, *mbn2*, *S2-DRSC*, *S2R+*, *S3*, *Sg4*, *W2* and *S1*) expressed *roX1* and/or *roX2* at male levels, which was again consistent with the deduced sex. The expression of *msl-2*, *tra*, and *Sxl* were also consistent with sex karyotype. Overall, the cell lines with a X:A >0.75 showed female expression, while those with a ratio of <0.75 showed male expression (*P* < 0.01, *t*-test); however, there was some ambiguity. For example, *D9* expressed intermediate levels of *roX1*, male levels of *msl-2* and female *tra*. We suggest that in the majority of cases X chromosome karyotype is the result of the sex of the source animals, but where karyotype and sex differentiation status are ambiguous, the X chromosome copy number may be due to gains/losses during culture.

**Table 2:** **Sex chromosomes and sex-biased expression**

Cell line ^*^	X:A ^a^	Y:A ^b^	Gene expression levels (FPKM) ^c^				Splicing events (PSI) ^d^	
			roX1	roX2	msl-2	traF	tra	Sxl
Kc167	0.94	0.00	0.41^‡^	1.17	1.94^‡^	9.04^‡^	0.22^‡^	0.00^‡^
1182-4H	0.95	0.00	5.19^‡^	0.11^‡^	3.75	16.67^‡^	0.52	0.04
D8	1.01	0.00	0.77^‡^	0.22^‡^	3.64	7.99	0.35	0.13
D16-c3	0.87	0.00	2.88^‡^	0.00^‡^	5.94^†^	16.75^‡^	0.38	0.29
D17-c3	0.84	0.00	0.23^‡^	0.13^‡^	6.79^†^	6.93	0.74	0.44
D9	0.86	0.00	66.98	8.82	14.34^†^	6.35	0.87	0.86
D4-c1	0.56	0.00	70.44	1.48	10.18^†^	0.32^†^	0.99	0.75
BG3-c2	0.56	0.63	0.16^‡^	29.65^†^	19.79^†^	0.54^†^	1.00^†^	1.00^†^
Cl.8	0.50	0.34	212.07^†^	38.37^†^	20.95^†^	0.54^†^	1.00^†^	1.00^†^
D20-c5	0.53	0.00	19.06	4.94	11.13^†^	0.00^†^	1.00^†^	1.00^†^
L1	0.54	0.00	96.50	7.73	24.10^†^	0.00^†^	1.00^†^	1.00^†^
mbn2	0.61	0.00	156.02^†^	11.54	22.64^†^	0.00^†^	1.00^†^	1.00^†^
S2-DRSC	0.55	0.01	8.17	51.43^†^	16.08^†^	0.00^†^	1.00^†^	1.00^†^
S2R	0.68	0.00	0.00^‡^	29.60^†^	13.27^†^	0.48^†^	1.00^†^	1.00^†^
S3	0.53	0.00	6.13^‡^	11.42	18.75^†^	0.00^†^	1.00^†^	1.00^†^
Sg4	0.54	0.00	106.46	18.82^†^	29.44^†^	0.00^†^	1.00^†^	1.00^†^
W2	0.55	0.04	60.20	2.99	12.93^†^	1.65	1.00^†^	1.00^†^
S1	0.52	0.24	198.00^†^	1.05	18.26^†^	0.00^†^	1.00^†^	NA

^a,b^X or Y chromosome to Autosome ratio (mapped DNA density).

^c^Expression levels of sex-specific genes. Expression levels are FPKM (fragments per kilobase per million reads) values.

^d^Levels of splicing events are summarized. PSI, proportion spliced in. PSI values close to 1 represent male-like splicing, and PSI values close to 0 represent female-like splicing.

^*^ *D20-c2* cell line: 0.53 for X:A, 0 for Y:A ratios (no RNA-Seq result).

^†,‡^Male or female characteristics, respectively, that are determined based on RNA-Seq analyses of 100 different fly lines (whole animals, *P* < 0.05, one sampled *t*-test).

Interestingly, both functionally redundant *roX* genes were expressed in whole adult males (not shown), while in the cell lines, sometimes only one *roX* gene was highly expressed. To determine if expression of a single *roX* gene was sufficient for MSL-complex-mediated dosage compensation, we measured X chromosome gene expression relative to autosomes. Overall transcript levels from genes from the X chromosomes in the cells that expressed *roX* genes at male levels were not significantly different from those of autosomes (*P* > 0.25 for all cell lines, *t*-test), suggesting that having a single *roX* is sufficient for normal X chromosome dosage compensation in these cell lines.

We observed frequent loss of the Y chromosome from the male cell lines. The *D. melanogaster* Y chromosome is not currently assembled, but some Y-chromosome genes are known. DNA-Seq reads were mapped on the Y chromosome (chrYHet) in a minority of the male cell lines (*BG3-c2*, *Cl.8*, *S1*, and *W2*) and we observed Y chromosomes by cytology in *BG3-c2*, *Cl.8* and *S1* lines (Additional file 1). The failure to map reads to Y chromosomes in the other male lines (*D20-c5*, *L1*, *mbn2*, *S2-DSRC*, *S2R+*, *S3*, *Sg4*) was also consistent with karyograms and reflects loss of Y chromosomes (Additional file 1). The Y chromosome bears only a few fertility genes (X/0 flies are sterile males) that should be of little consequence outside the germline. Frequent loss suggests that there is little selective pressure to maintain a Y in tissue culture cells.

**Figure 3:**
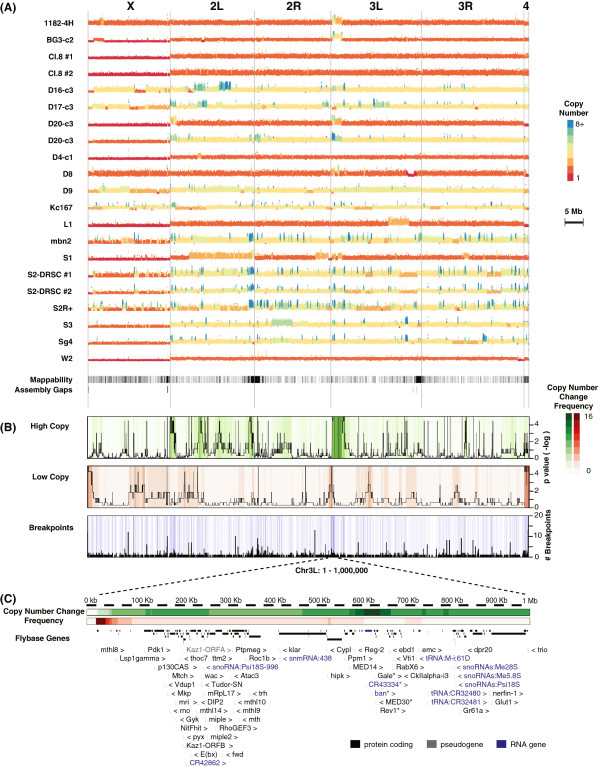
**DNA copy numbers. (A)** Plots of mapped DNA read density along the genome. Deduced copy number is indicated by color (see key). **(B)** Heatmaps display how many cell lines have increased (green) or decreased (red) copy number. Black lines in the first two rows show significance. Blue lines indicate breakpoints. Black in the bottom row shows the number of breakpoints shared by the 19 cell lines. **(C)** A zoomed-in map of the sub-telomeric region (1 Mb) of chromosome 3 L. Asterisks: genes within the highly duplicated regions. Genes with little or no functional information (‘CG’ names) were omitted for brevity.

Lastly, we observed widespread loss/gain of the short (approximately 1.4 Mb) fourth chromosome in cell lines by both DNA-Seq and cytology (Figure 3A; Additional file 1). The number of fourth chromosomes was variable within cell lines as well. As an illustration, in *Cl.8* cells where overall genome structure is relatively intact diploidy, the number of fourth chromosomes varied from 0 to 3. This observation was also supported by DNA-Seq results, which demonstrated clear decrease of copy number (combined *P* < 1.0e-11, false discovery rate (FDR)-corrected permutation test).

### Segmental and focal copy number changes

We observed frequent sub-chromosomal copy number changes (Figure 3A; Additional file 3). Some of the larger departures from ploidy were also identifiable in the karyograms. For example, mitotic spreads of *S1* cells exhibited an acrocentric chromosome that looked like the left arm of chromosome 2 (‘2r’ in Additional file 1), which was reflected in DNA-Seq data as extended high copy number block. However, most of the focal changes were submicroscopic in the low megabase range. Collectively, we observed more increases of copy number (1,702) than decreases (388). On average, 12.9% of the haploid genome was duplicated, or gained, while 6.3% was deleted, or lost; 95% of the copy number blocks were shorter than 0.8 Mb (median = 37 kb) in the case of increased copy and 1.8 Mb (median = 97 kb) in the case of decreased copy.

DNA-Seq data showed that genome structure was cell line-specific. For example, in *Cl.8* cells we observed few copy number changes, which were spread over multiple small segments covering only 0.88% of the genome. In contrast, in *S2-DRSC* and *Kc167* cells, we observed copy number changes for >30% of the genome. Interestingly, *Kc167* cells had more low copy number regions than high copy number regions, while *S2-DRSC* had more high copy number regions than low copy number regions. These data indicate that there are fundamentally different routes to a highly rearranged genomic state.

While the overall genome structures were cell line-specific, we did observe regions of recurrent copy number change. While some of the cell lines (for example, *S2R +* and *S2-DRSC*) are derived from a single ancestral cell line and differ by divergence, the majority of the cell lines were isolated independently, suggesting that similarities in genome structure occurred by convergent evolution under constant selection for growth in culture. Our investigation revealed 89 regions of the genome covering a total of approximately 9.3 Mb showing strong enrichment for increased copy number (Figure 3B; *P* < 0.05, FDR-corrected permutation test). Among those segments, 51 regions were longer than 5 kb. We also found 19 regions covering approximately 2.9 Mb with significant enrichment for decreases in copy number; 14 of these regions were longer than 5 kb. Driver genes promoting growth in culture may be located in these regions.

We examined regions of recurrent copy number change more closely to identify some candidate drivers. As an illustration, duplications of sub-telomeric regions of chromosome 3 L (approximately 3 Mb) were found in 10/19 cell lines (combined *P* < 1.0e-16, FDR-corrected permutation test). The most overlapping segment within this region was a duplication region of approximately 30 kb. There are six annotated genes in this core duplicated segment (Figure 3C, asterisks): *CR43334* (pri-RNA for *bantam*), *UDP-galactose 4′-epimerase* (*Gale*), *CG3402*, *Mediator complex subunit 30* and *UV-revertible gene 1* (*Rev1*). When we asked if any of these specific genes showed increased copy number in the other cell lines, even if segmental structure was lacking, we found that *CR43334* and *Rev1* had higher copy numbers in five additional cell lines. As another example, an approximately 19 kb duplication region in chromosome 2 L was found in 10 different cell lines (combined *P* < 1.0e-17). This region included only one gene, *PDGF- and VEGF-receptor related* (*Pvr*), suggesting that copy number for this gene is highly selected for in cell culture. If genes in these recurrent copy number increase regions were drivers, then we would expect that they would be expressed in the cells. Indeed, pri-*bantam* and *Pvr* genes were highly expressed in the cell lines (Additional file 4).

### Mechanisms generating segmental and focal copy number changes

Creation of common copy number changes would be facilitated by repeated breakage at ‘hot spots’ in the genome due to regions of microhomology or longer stretches due to structures such as inserted transposons. In the absence of selection, the extant breakpoint distribution would map the positions of such hot spots. We mapped breakpoints by examining read-count fluctuations in every 1 kb window over the genome to identify 2,411 locations with breaks in at least one of the 19 cell lines (Figure 3B; Additional file 3). Among these breakpoints, we discovered 51 hotspots of copy number discontinuity in the same 1 kb window (*P* = 5.00e-06, permutation test). This suggests that there are regions in the genome that suffer frequent breaks in tissue-culture cells. Investigation of hot spots revealed 18 containing long terminal repeats (LTRs) or long interspersed elements (LINEs) in the reference assembly, and an additional 9 regions showed simple DNA repeats within the 1 kb (±1 kb) windows. These observations are consistent with reports of overrepresentation of sequence repeats at copy number breakpoints [13], and with the suggested roles of transposable elements in the formation of copy number variants [46, 47]. For the recurrent copy number change regions, we observed a broad regional enrichment for breakpoints (*P* = 4.07e-10, Fisher’s exact test), but not precise locations. These data suggest that there were both structural features in the genome that promoted generation of copy number changes and selection that determined which copy number changes were retained.

### Expression and DNA/chromatin binding profiles in relation to copy number

**Figure 4:**
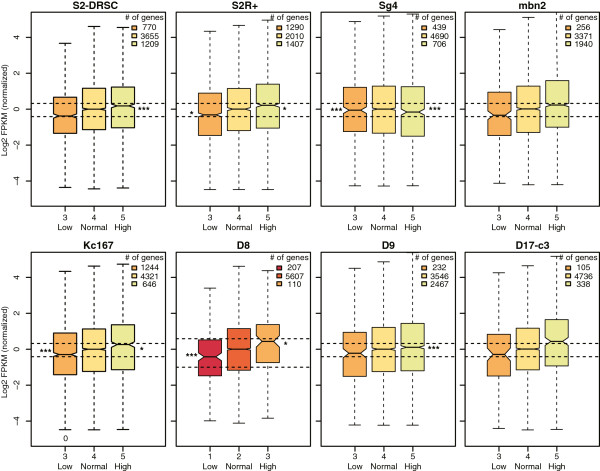
**Copy number and expression.** RNA-Seq analysis of *S2-DRSC*, *S2R+*, *Sg4*, *mbn2*, *Kc167*, *D8*, *D9* and *D17-c2* cells. Boxplots show interquartile ranges of the distribution of FPKM (fragments per kilobase per million reads) values of expressed genes (FPKM >1) for different copy number classes in the indicated lines. The number of genes in each class is shown. All FPKM values are centered to have the median of normal copy number gene expression as 0. Top, middle, and bottom lines of boxes correspond to upper quartile (Q3), median, and lower quartile (Q1) in the distribution, respectively. Notches show the 95% confidence interval of each median. Whiskers indicate the maximum, or minimum, value that is still within 1.5 times of interquartile distance (Q3 - Q1) from Q3 or Q1, respectively. Horizontal dashed lines indicate the expected FPKM values based on a one-to-one relationship between gene dose and expression. Asterisks display *P*-values, determined by Mann-Whitney *U* test (*P < 0.05, **P < 0.01, ***P < 0.001).

If copy number changes have a role in cellular fitness, the effect might be mediated by altered gene expression. We therefore examined the relationship between gene dose and expression in 8 cell lines that had more than 100 expressed genes in high or low copy number segments (Figure 4). In seven cell lines (*S2-DRSC*, *S2R+*, *mbn2*, *Kc167*, *D8*, *D9* and *D17-c3*) mRNA level was positively correlated with gene dose. There was no correlation between gene expression and gene dose in *Sg4* cells. Even in the cases where the correlation was positive, the correlation was usually not linear, as has been previously observed [31]. In most lines, we observed decreased expression per copy of high copy number genes (*P* < 0.05, Mann-Whitney *U* test). Similarly, overall gene expression of the low copy number genes was moderately higher than expected on a per copy basis (Figure 4). This sublinear relationship is evidence for a transcriptional dampening effect.

**Figure 5:**
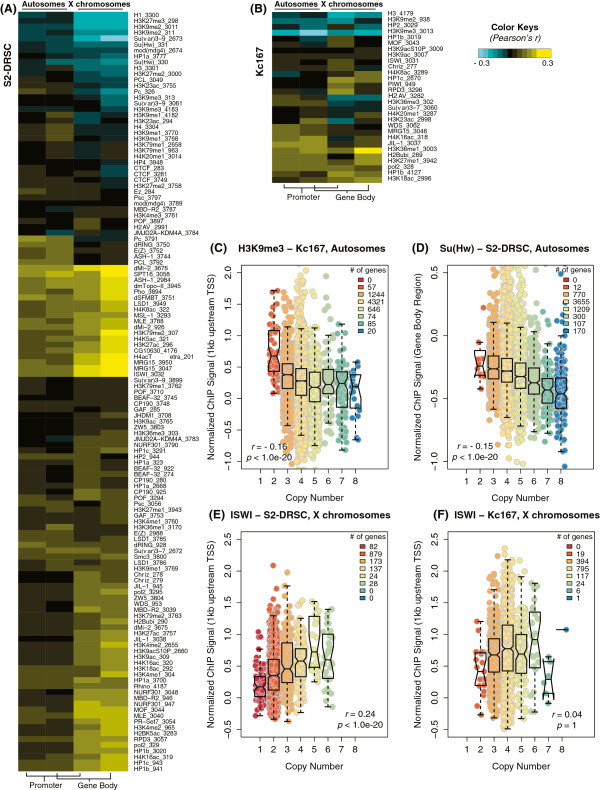
**Copy numbers and chromatin immunoprecipitation. (A,B)** A heatmap that summarizes correlation between copy numbers and chromatin immunoprecipitation (ChIP) signals of expressed genes in *S2-DRSC* **(A)** or *Kc167* **(B)** cell lines. Target proteins for ChIP and modENCODE submission numbers are listed (right side). Columns show autosomal promoter regions (1 kb upstream of transcription start) and gene body regions as indicated. **(C,D)** ChIP signals of H3K9me2 **(C)** and SU(HW) **(D)** at autosome gene bodies are displayed against different copy number classes as boxplots (*S2-DRSC* cells). Top, middle, and bottom lines of boxes for upper quartile, median, and lower quartile points, respectively. Notches indicate the 95% confidence interval of each median and whiskers display the maximum, or minimum, value within the range of 1.5 times of interquartile distance, respectively. Dots display individual genes within different copy number classes. Pearson’s correlation for *r* and its significance (*P*-value). **(E,F)** ISWI ChIP signal analyzed for X chromosome gene bodies in a male (*S2-DRSC*; **E**) and a female (*Kc167*; **F**) cell line. TSS, transcription start site.

The transcriptional response to gene copy number could be gene-specific or dose-specific. A dose-specific compensation system might be expected to result in a global change to chromatin structure corresponding to copy number segments. There is precedent for such dose-specific modifications of X and fourth chromosomes. For example, the modENCODE chromatin structure analysis of *S2-DRSC* cells clearly shows differences between X and autosomal chromatin using any of a host of histone modification or binding of chromatin-associated proteins (Figure 5). This is consistent with the global regulation of the X in these male cells by the MSL complex and perhaps other regulators [27, 28].

To determine if there was a chromatin signature for copy number, we asked if there were histone modification marks or occupancy sites that correlated with copy number classes in 232 modENCODE ChIP-chip datasets from *S2-DRSC*, *Kc167*, *BG3-c2* and *Cl.8* cells. We observed only a few weak correlations (|*r*| = 0.1 to 0.3), restricted to histone H3K9 di- and tri-methylation marks, and their related proteins (Figure 5), Suppressor of Hairy wing (SU(HW)), and Imitation SWI (ISWI). These correlations were slightly stronger for expressed genes. Interestingly, ISWI binding correlated with copy number on the X chromosome of male *S2-DRSC* cells, but not female *Kc167* cell X chromosomes. ISWI binding did not correlate with autosomes of either line. This localization on the X is consistent with the known role of ISWI protein in X chromosome structure, as ISWI mutant phenotypes include cytologically visible ‘loose’ X chromatin only in males [48, 49]. We found that histone H3K9me2 and me3 marks were negatively correlated with gene copy numbers in all four tested cell lines on all chromosomes. The histone H3K9 methyltransferase, Suppressor of variegation 3-9 (SU(VAR)3-9), showed the same pattern of binding, strongly supporting the idea that H3K9 methylation is a copy number-dependent mark. H3K9me2 and H3K9me3 epigenetic marks are associated with transcriptional repression [50]. SU(HW) functions in chromatin organization and is best known for preventing productive enhancer promoter interaction. Thus, the relationship is the opposite that one would expect if H3K9me2, H3K9me3, and SU(HW) were responsible for the reduced expression per copy we observed when copy number was increased. These results are more consistent with selection to drive down expression of these regions by both reduced copy number and transcriptionally unfavorable chromatin structure.

### Pathway coherence

**Figure 6:**
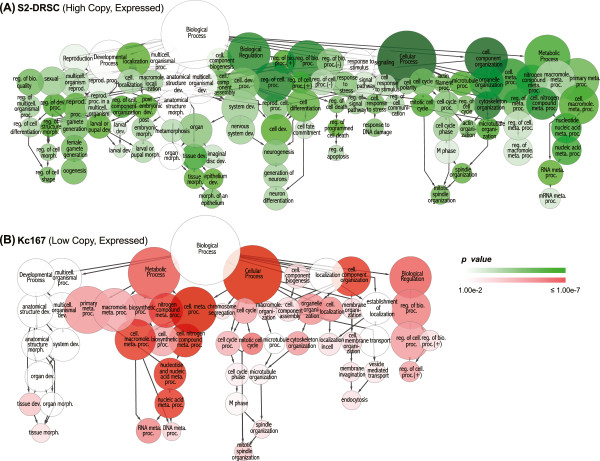
**Gene Ontology and copy number in** ***S2-DRSC*** **and** ***Kc167*** **cells. (A)** ‘Biological processes’ sub-ontology of overrepresented genes in *S2-DRSC* cells as a hierarchical structure. Circle size corresponds to relative enrichment of the term in GO categories. Circle colors represent *P*-values (Holm-Bonferroni corrected hypergeometric test). **(B)** GO enrichment of genes in low copy number segments of *Kc167* cells. Please note that both *S2-DRSC* low and *Kc167* high copy number genes are not significantly enriched in specific GO categories.

If there has been selection for particular advantageous copy number configurations in the cell lines, then this should result in a coherent pattern of events in terms of specific cellular activities such as growth control. As a first pass analytical tool, we performed Gene Ontology (GO) term enrichment analysis to determine if copy number changes were associated with particular functions (Figure 6; Additional file 4). Tissue culture cells have no obvious need for many of the functions associated with the complex interactions between tissues and organs in a whole organism and should not undergo terminal differentiation. Indeed, we found that genes with differentiation functions were randomly found in copy number change regions but were enriched in low copy number regions in *Kc167* cells (*P* < 0.001, Holm-Bonferroni corrected hypergeometric test). Additionally, we found increased copy numbers of genes encoding members of the dREAM complex in *S2-DRSC*, *mbn2*, *S1* and *S2R +* cells. The dREAM complex represses differentiation-specific gene expression [51, 52], consistent with selection for copy number changes minimizing differentiation.

The most significant associations (*P* < 0.001) between copy number class and function were with genes having cell cycle, metabolic, or reproduction-related GO terms (reproduction-related categories contain many of the cell cycle genes due to the high rates of cell divisions in the germline relative to somatic cells in adult *Drosophila*). Interestingly, genes with cell cycle-related functions were enriched in both high copy number regions in *S2-DRSC* and low copy regions in *Kc167* cells (*P* < 0.001 for both). The context of this dichotomy was informative. Genes with high copy numbers in *S2-DRSC* cells included *Ras oncogene at 85D*, *string*, *Cyclin D*, *cdc2*, and other positive regulators of cell cycle progression, or mitotic entry. These data suggest selection for growth occurred in *S2-DRSC* cells. In contrast, tumor suppressor genes, and negative regulators of cell cycle, including *Retinoblastoma-family protein* (*Rbf*), *Breast cancer 2 early onset homolog* (*Brca2*), and *wee*, were preferentially found in the low copy number regions of *Kc167* cells, suggesting that inhibitors of cell growth were selected against in *Kc167* cells. Thus, both the high copy number and low copy number events can be explained by selection for proliferation.

### Compensatory copy number changes

Copy number changes in adult *Drosophila* result in propagation of transcriptional effects into the rest of the genome [53]. As these events can destabilize gene balance in pathways and complexes, we hypothesized that compensatory copy number changes might boost fitness. To examine this possibility, we asked if genes have undergone copy number changes to maintain protein-protein complex stoichiometry by overlaying copy number information of *S2R +* cells onto a physical protein interaction network that was built from complexes isolated from the same cell line [54].

**Figure 7:**
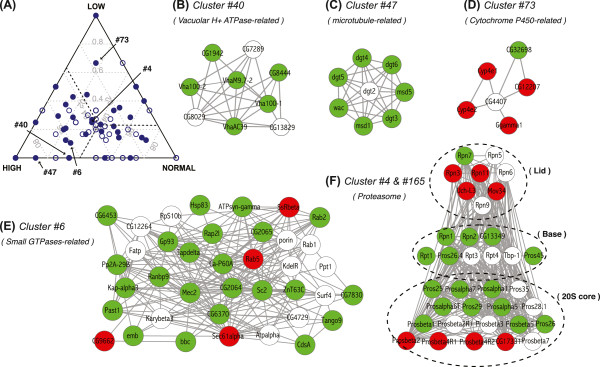
**Copy number and physical interaction networks. (A)** A ternary plot that displays fractions of high, normal, and low copy number genes that encode complexes in *Drosophila* protein-protein interaction networks. Each point corresponds to a protein complex or a cluster. Distances from the three apexes in the triangle indicate fraction of cluster members from a given copy number class. Dashed lines indicate expected portion of each copy number class based on a random distribution of *S2R +* cell line copy numbers. Complexes where copy number composition is significantly different from the expected ratio (*P* < 0.05, hypergeometric test) are filled in blue. **(B-F)** Protein interaction networks described and labeled in **(A)**. Green, high copy gene products; red, low; white, normal. For **(F)**, six proteins whose associations with the proteasome parts are not clear in the literature were omitted.

There were 142 protein-protein interaction networks that contained at least one gene product encoded from copy number change regions (Figure 7A). Among these, we identified 84 complexes that had >90% co-occurrence of copy number change in the same direction at the gene level (*P* = 0.041, permutation test). These copy number changes were not due to passenger effects as stoichiometry-preserving changes in copy number were still evident after filtering for nearby genes (*P* = 0.03). Examples included the genes encoding Vacuolar H^+^ ATPase (*P* = 0.017, hypergeometric test) and Dim γ-tubulin (DGT) complexes (*P* = 0.004), where members were among high copy number genes (Figure 7B,C). For both complexes, genes encoding their components were spread on five different chromosome arms with only a pair of genes showing <0.5 Mb proximity, indicating that the co-associations are not due to simple physical proximity in the genome. We also identified complexes where the encoding genes were in low copy, such as a Cytochrome P450-related complex (*P* = 0.001; Figure 7D). We found correlated copy number changes even for very large complexes, such as the small GTPase related-complex (cluster 6), which has 38 proteins. Twenty-four of the loci encoding cluster 6 members were present at high copy (Figure 7E; *P* = 5e-04). By examining complexes where we failed to score a simple correlation, we uncovered more complicated patterns where sub-components of the complex show correlated and anti-correlated copy number changes. A good illustration is the proteasome (Figure 7F). While the overall composition was consistent with genome-wide copy number levels, we found that genes encoding the lid of the regulatory 19S subunit showed coherent copy number reduction in *S2R +* cells (*P* = 0.015, hypergeometric test). In contrast, proteins composing the base and alpha-type subunits of the 20S core were dominated by copy number gains (*P* = 0.017 and 0.014, respectively). This suggests that the actual occurrence of coherent copy number changes among genes encoding protein complex members may be higher than what we report here.

## Discussion

### Copy number and cell line evolution

In our study, we provide copy number maps for 19 cell lines that display copy number differences relative to the *Drosophila* reference genome. Some cell lines, such as *Cl.8*, *D4-c1*, and *W2*, have relatively intact genomes. In contrast, the cell lines that are more widely used in the *Drosophila* community, such as *S2-DRSC*, *Kc167*, and *S2R+*, show extensive copy number change for >40% of the genome. Some of copy number differences might simply reflect genome structures in the source animal. For example, we have observed similar genome structures for *D20-c2* and *D20-c5*, which were generated from the same original animals. Retention of the source genome structure would suggest that copy number change can be quite stable during cell passage, although many of the cell lines were derived from the same genotype of flies and have been rarely used since freezing. We also inferred change of genome structure over time. For example, we observed structural discrepancies between *S2-DRSC* and *S2R +* cell lines, even though these cell lines were derived from the original *S2* cells circa 1972 [39]. While *S2-DRSC* has been grown very extensively in multiple labs since it was established, *S2R +* spent more than 25 years in a freezer, and has been grown sparingly in the 15 years since [40]. The approximately 32% difference in copy number between these two lines indicates that the long period of *in vitro* culture of *S2* cells contributed to the changes. Unfortunately, records for passages and transfers of cell lines among labs are anecdotal at best, so we cannot estimate change per passage. Nevertheless, cell line genome structure suggests that some elements of initial genotype are conserved, while most copy number changes are acquired. Locations of many copy number changes were shared among several cell lines, even those with clearly different sources, indicating that recurrent copy number changes have occurred.

Recurrence depends on a combination of biased generation of rearrangements and selection for the resulting copy number changes. Syntenic blocks reveal patterns of genome structure in *Drosophila*[55, 56]. However, the occurrence of copy number discontinuity was only marginally biased with respect to syntenic blocks (about 10% more intra-syntenic breaks than inter-syntenic disruption). Furthermore, the breakpoints we identified demonstrated poor overlap with common fragile sites that are induced by aphidicolin treatment [57]. Similarly, comparison of recurrently low copy regions in the cell lines to the previously reported 65 regions where DNA replication was significantly repressed in salivary glands [58] identified only three regions (all sub-telomeric) that were at least partially overlapping. While structural factors are prerequisites for breaks, repair, and recombination, the observation that there are shared copy number changes, including potentially useful driver genes, suggests that copy number evolution is functionally constrained, as has been suggested for copy number polymorphism patterns at the organismal level [59–61]. Interestingly, except for regions where unambiguous mapping of reads is complicated by low sequence complexity (which may contribute to copy number change), we were not able to find any significant overlap between regions of copy number polymorphism in *Drosophila* animal populations and the copy number regions we identified in the cell lines. This suggests that the combination of hot spots for breaks and selective forces are distinct at the animal and cellular levels.

### Gene dosage effects and compensation

The amount of transcript produced from genes with a given copy number is a function of both the gene dose and secondary changes in the rest of the genome, including feedback regulation and buffering due to kinetics [62]. The sum of these trans-effects in gene networks can antagonize the dosage effect and result in gene-specific dosage compensation [63]. We observed clear dosage effects in 19 cell lines, and the response varied from compensated, to sub-linear buffering, to nearly linear relationships between dose and expression. Previous genome-wide expression studies on *Drosophila S2-DRSC* cells [31], adult *Drosophila*[53, 64, 65], and human cells [66] have shown sub-linear relationships between copy number and expression. Two models have been proposed for the observed dosage effects and partial compensation in *Drosophila*[31, 53, 64]. The first model proposes that there is a variable gene-by-gene response to copy number, which is mediated by regulatory feedback systems. The variable dose/response characteristics we observed in this study support this model. The second model proposes the existence of a copy number recognition system, analogous to MSL and POF, which uniformly adjust expression of genes with a given altered dose. While we did find evidence for dose-specific histone marks, the pattern is difficult to reconcile with a global compensatory response to copy number. The observed modifications would be expected to exacerbate the dose effect, not enhance dosage compensation. It seems likely that both transcriptional repression and reduced copy number of these regions are selected to increase cellular fitness.

### Apoptosis

One of the more striking observations suggests that pro-survival gene copy number has been under heavy selection. For example, almost 80% of the cell lines acquired additional copies of the pri-*bantam* gene, and there was higher expression of the *bantam* microRNA (miRNA) in those cell lines. *bantam* is an anti-apoptotic miRNA that suppresses the pro-apoptotic function of *Wrinkled* (a.k.a. *hid*) and prevents proliferation-induced cell death [67]. Indeed, *bantam* was the most abundant miRNA in 25 cell lines, which were surveyed in the small-RNA component of modENCODE [68]. This strongly suggests that additional copies of the *bantam* gene are drivers providing selective advantages to cell lines.

Supporting the apoptosis suppression hypothesis, we also discovered that the platelet-derived growth factor (PDGF)/vascular endothelial growth factor (VEGF) receptor-encoding *Pvr* gene is duplicated in 10 cell lines. *Pvr* also promotes anti-apoptotic survival, as loss of *Pvr* causes apoptosis and reduces the number of hemocytes in *Drosophila* embryos, which can be rescued by the pan-caspase inhibitor p35 [69]. *Pvr* and the PDGF/VEGF receptor ligand encoding *PDGF- and VEGF-related factor 2* genes are highly expressed in the cell lines where the copy numbers of those genes have increased [41]. This suggests that cell lines select for anti-apoptotic activities. Consistent with this suggestion, RNA interference screening of viability and growth-related genes has demonstrated that knockdown of *Pvr* reduces viability of cells and decreased mitotic as well as cytokinetic indexes in *S2*, *S2R+*, and *Kc167* cells [70–72].

Support for copy number modification of apoptosis responses is extensive. In addition to *bantam* and *Pvr*, many genes involved in the JNK pathway [73] showed changes in copy number in the *S2-DRSC* and *Kc167* cell lines. For example, *basket* (encoding JNK) was located in a duplicated region in *S2-DRSC* cells. In contrast, *Kc167* had fewer copies of *puckered* (encoding mitogen-activated protein kinase phosphatase) that functions to negatively regulate JNK activity. Finally, the *kayak* gene (encoding FOS), a downstream target of JNK, was found at a highly duplicated region of chromosome 3R (10 copies). These conditions of potentially high JNK activity in both cell lines would induce apoptosis in normal cells [74, 75]. However, it is known that the same condition may promote cell growth and proliferation when the caspase cascade is compromised [75]. Thus, high JNK pathway activity would be advantageous to cells in culture only if caspase pathway activity was reduced.

*Drosophila* has two important initiator caspases [76], Death-related Ced-3/Nedd-2 like protein (DREDD) and Nedd-2 like caspase (NC). The genes for both of them were found in low copy number regions in *S2-DRSC* and *Kc167* cells. The inhibition of the caspase pathways can also be mediated by inhibitor of apoptosis proteins (IAPs). *Drosophila* has at least two genes that encodes IAP-like proteins and inhibit caspases; *thread* (encoding IAP1) and *Inhibitor of Apoptosis 2* (*Iap2*) [77]. While they are not clustered on the genome (chromosome 3 L and 2R, respectively), both of them were found in high copy number regions in *S2-DRSC* cells. In combination with JNK, these copy number changes might help cells grow in culture while minimizing apoptosis. Indeed, RNA interference-mediated depletion of *thread* or *Iap2* results in reduced cell viability and increased apoptosis in *S2*, *S2R+*, or *Kc167* cells [70, 78, 79], which is suppressed by inhibition of caspase cascade activation in *S2* cells [79]. These observations are reminiscent of the situation in cancer cells, where the copy number of anti-apoptotic genes are overrepresented and pro-apoptotic genes are underrepresented [17]. This suggests shared roles of copy number in these cell-level natural selection progressions and underscores the advantages of *Drosophila* cell lines in the study of tumorigenesis.

### Cell cycle and repair

Copy numbers of cell cycle-regulator genes may also contribute to the proliferative nature of the cell lines. Positive regulators of the cell cycle, such as the *Cyclin E*, or *string* genes, were located in high copy regions in 4 different cell lines and were never represented in low copy number segments among 19 cell lines. In contrast, well-known negative regulators of the cell cycle, such as *Rbf* and *Brca2*, were often found in low copy number regions, and never found in high copy number regions. In addition to the cell cycle, or apoptosis-related genes, frequent duplication (15 cell lines) of *Rev1*, which is near *bantam*, is also of note. Yeast Rev1p is required for mutagenic bypass to help repair a range of DNA lesions [80]. Similarly, *Drosophila* REV1 regulates a switch between highly processive DNA polymerases to lesion bypassing polymerases, such as DNA polymerase *zeta* and *eta*[81]. This raises the possibility that overrepresentation of the *Rev1* gene may contribute to hypermutability of the cell lines. However, it is also possible that *Rev1* copy number is simply driven by linkage to *bantam* as a passenger.

## Conclusions

**Figure 8:**
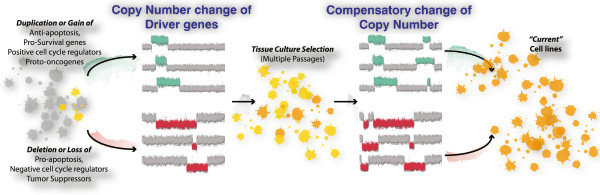
**A schematic model of copy number evolution.** At an early stage of cell line establishment, cells that acquired ‘advantageous’ copy number changes would be selected due to the dosage effect of potential driver genes. We suggest that these included increased copy number for anti-apoptosis, or pro-survival genes as well as decreased copy number of pro-apoptotic or tumor suppressor genes. Further culture passages selected cells with more optimized genome structure that restored genic stoichiometric imbalance caused by drivers and especially passenger copy number changes.

Our results strongly suggest that copy number is a potent way for cells to evolve to culture conditions (Figure 8). We suggest a two-step process, where copy number changes in critical genes increase growth and survival, followed by refined selection to restore genic balance. While very specific changes in copy number of driver mutations might maximize growth, these changes in copy number usually extend into neighboring genes. This imbalance has the potential to destabilize protein complexes. That mutations are co-selected to maintain gene balance is an old idea [82, 83], and our work supports this idea.

It seems likely that copy number changes are a generic feature of tissue culture cells and tumors, which share an uninhibited growth phenotype. Genomic aberrations, sustaining a proliferative state, and resisting cell death are hallmarks of cancer [84, 85]. The tantalizing links between copy number changes observed in tumors and *Drosophila* cell lines suggest that the power of *Drosophila* genetics can be applied to human diseases with copy number etiology.

## Materials and methods

### Cell culture and library preparation

The cell lines used for DNA resequencing and RNA-Seq were grown and harvested as described [41], except that *Kc167* cells were cultured in the serum-free medium CCM-3 (HyClone, Logan, UT, USA) rather than in Schneider’s medium with 10% serum, and *S2-DRSC* and *BG3-c2* were cultured in M3 + BPYE in place of Schneider’s medium. Cells were harvested at plateau for DNA extraction.

For DNA libraries, 1.5 × 10^7^ cells were rinsed in phosphate-buffered saline and incubated with 2 mg of Proteinase K (Amresco 0706, Solon, OH, USA) for 2 hours at 37°C, phenol-chloroform extracted, and ethanol precipitated. Resuspended nucleic acid was digested with 50 μg of RNaseA (Amresco 0675) for 1 hour at 37°C. Final ethanol precipitation was performed with 0.3 M (final) NaOAc. Resuspended DNA was fragmented to less than 800 bp by sonication. Libraries were prepared as described (‘Preparing samples for sequencing genomic DNA, part # 11251892’; Illumina, San Diego, CA, USA), with the exception of an additional gel extraction (size select for 150 to 200 bp) after the PCR step (see modENCODE website for details [86]).

DNA resequencing of *BG3-c2*, *Cl.8*, *S2-DRSC*, and *Kc167* was performed with the Illumina-based short-read sequencing platform. They were run for 36 cycles on a GAII or HiSeq 2000 (Illumina). The other cell lines used in this study, as well as an independent set of *Cl.8*, were sequenced to have either 76 or 100 bp paired-end reads on a GAII or HiSeq 2000 (*1182-4H*, *Cl.8+*, *D16-c3*, *D17-c3*, *D20-c2*, *D20-c5*, *D4-c1*, *D8*, *D9*, *L1*, *mbn2*, *S1*, *S2R+*, *S3*, *Sg4*, and *W2*). We also re-analyzed *S2-DRSC* sequencing data from a previous study.

For RNA libraries, the extraction of total RNA from the cell lines was previously described [41]. RNA-Seq libraries were prepared as in [87], and a further detailed protocol can be found from modENCODE DCC. The sequencing was performed on Illumina platforms (GAII or HiSeq200). RNA-Seq of *BG3-c2*, *Cl.8*, *S2-DRSC*, and *Kc167* was performed as unstranded paired-end sequencing with 37 bp read-length. The other cell lines were paired-end sequenced to 76, 78, 100 or 108 bp read-length in a strand-specific manner (*1182-4H*, *D16-c3*, *D17-c3*, *D20-c5*, *D4-c1*, *D8*, *D9*, *Kc167*, *L1*, *mbn2*, *S1*, *S2R+*, *S3*, *Sg4*, and *W2*).

### Previous modENCODE datasets

ChIP-chip datasets were from the modENCODE Data Coordination Center (DCC) [86] and are also available in the NCBI Short Read Archive (SRA). We used a total of 232 datasets of ChIP-chip as well as nucleosome profiling on microarrays from modENCODE [88]. See te Data access section below for the list of all datasets used.

### Data processing and copy number calling

We aligned both DNA and RNA sequencing data to the reference *D. melanogaster* genome that we obtained from UCSC genome browser (dm3, which corresponds to Berkeley Drosophila Genome Project release 5; excluding chrUextra). We mapped with Bowtie 0.12.8 for reads shorter than 50 bp, or Bowtie2 2.0.2 for longer read lengths [89, 90]. We allowed up to two mismatches from short read data with unique mapping (-v 2 -m 1 parameters) for Bowtie. We used Bowtie2 in its end-to-end mode with the ‘sensitive’ preset option.

The alignment results were used to obtain ratiometric DNA densities in 1 kb windows using FREEC 5.7 [91]. For segments defined by the LASSO method (Least Absolute Shrinkage and Selection Operator), the median DNA content of each segment was given to all 1 kb windows. The mean of DNA read density was set as 1 and other bins were represented as ratios based on the mean. We used clustering analysis to estimate different DNA content levels. The agglomerative nesting algorithm (AGNES) was used with R program language and its package ‘cluster’ [92]. Any DNA density ratios that had less than 500 bins (=500 kb) were excluded. We set minimum dissimilarity between cluster centers (=interval between peaks) as 0.167, which is expected from hexaploidy. We counted possible numbers of ploidy levels from 0 (no DNA) to 1 (expected DNA density of the majority of the genome). This estimation was used as an input of FREEC to define baselines of copy number calling, except for *D20-c5*. We used tetraploid-baseline for *D20-c5*, from the karyogram. For *D9* and *mbn2* cell lines, we performed further calculations based on tetraploidy. When karyograms suggest a mixed population of diploid and tetraploid cells, we used our estimation from DNA-Seq as our baselines to account for the detectable copy number segments (*BG3-c2* and *D4-c1*, diploids; *D16-c3* and *D17-c3*, tetraploids).

Samtools v.0.1.18 [93] was used to determine X chromosome or Y chromosome to autosome ratios from DNA-Seq results. Mean coverage (Read length × Number of mapped reads/Haploid length of the reference genome) of X chromosomes and all autosomes was compared except for chrU in the reference genome. Scaffolds based on heterochromatic regions (chrXHet, chr2LHet, chr2RHet, chr3LHet, and chr3RHet) were not used except for chrYHet. To avoid the severe mappability issue on the Y chromosome (chrYHet), the Y:A ratios were obtained from a 5 kb region with no obvious DNA repeats (chrYHet:140,000-145,000).

We used the GEM mappability program (GEnome Multitool) packages to define regions with poor mappability, and marked any 1 kb windows with less than 90% mappability as unknown [94]. We generated different mappability profiles based on different lengths of short reads by allowing up to two mismatches. The minimum and the maximum of expected GC contents were set as 0.3 and 0.45, respectively, in FREEC. Gene copy numbers were assigned based on the gene model. We did not call copy numbers for genes with any 1 kb windows where copy number was not determined. When copy number change occurred within a gene, we chose the call for transcription start site.

To calculate significance of copy number changes among cell lines, we performed permutation tests. We randomly shuffled locations of 1 kb windows within a cell line genome-wide one million times to determine *P*-values of 1 kb window copy number changes. We adjusted *P*-values using the Benjamini-Hochberg method for the multiple hypothesis correction [95]. Stouffer’s method [96] was used to combine *P*-values where specific regions were described. Analysis of breakpoints was performed with custom scripts written in R. We used sequence for the breakpoints that were found from five or more cell lines to find potential motifs with the MEME suite (minimum motif length 2 bp, maximum 50 bp) [97].

For RNA-Seq analysis, we used Ensembl release 67 [98] of Flybase 5.39 [99] gene annotations. A minor alteration was made to remove antisense transcripts of *mod(mdg4)* since these caused errors in downstream analysis. RNA-Seq results were aligned to the genome using TopHat 2.0.6 [100]. TopHat runs on Bowtie, and therefore we selectively used either Bowtie or Bowtie2 based on the read lengths. Reads were uniquely mapped with a gene model provided (-g 1 -G parameters). We set 200 bp as inner distance between pairs and 40 bp for the minimum intron lengths (-r 200 -i 40). For experimental sets with 36 bp read-lengths, we additionally used the segment-length 16 option. We used Cufflinks 2.0.2 to calculate transcript abundance in FPKM (fragments per kilobase per million reads) based on the reference annotation (-G parameter) [101]. Option ‘-b’ was used to account for the random hexamer-based bias. For the results presented in this study, we used FPKM >1 as a cutoff for gene expression [102].

We analyzed sex-specific splicing events using Spanki 0.4.0 (splicing analysis kit) [103]. We used the quickjunc utility within Spanki, with alignment files generated by TopHat as input, to quantify splice junction coverage, requiring an anchor size of 8 bp. We defined pairwise splicing events with AStalavista [104] and used the spankisplice utility to identify splice junctions that compose mutually exclusive splice variants (inclusion and exclusion forms). For clarity in presenting results for differential splicing in sex-determination pathway components, we labeled the male/female predominant forms as the inclusion/exclusion forms, respectively. Each of these forms was then quantified with the average of their junction coverage. Proportion spliced in (PSI) for splicing events was calculated by dividing the junction coverage of the inclusion form by the sum of the inclusion and exclusion coverage. This yields a PSI value between 0 (predominance of the exclusion form) to 1 (predominance of the inclusion form). Results from RNA-Seq analysis of 200 different male and female flies are used to provide reference ranges of sex-specific gene expression and splicing events (HL, S Russell, and BO, unpublished).

ChIP signals from microarray datasets were based on normalized intensity ratio (M values) in wiggle format files. Areas under the wiggle histograms were calculated and normalized with the length of regions of interest using R. We determined ChIP signals for 1 kb upstream of transcription start and gene body regions separately. Pearson’s correlation was used to analyze the relationship between copy number and ChIP signal. We used r > 0.1 and *P* < 0.001 as a cutoff of correlation (r > 0.2 for X chromosome).

### Protein interaction network analysis and Gene Ontology study

Lists of genes in the protein-protein interaction network were from the Drosophila Protein interaction Map (DPIM) [54]. We used clusters with *P* < 0.01, and integrated copy number information (*S2R +* cell line) with an R script. Differences in the number of genes showing copy number change from the expected value were tested by Fisher’s hypergeometric test. Significance of the number of DPIM clusters with coherent copy number change was tested by permutation tests (1,000 times with no replacement). We used Cytoscape 2.8.3 to visualize networks [105]. To account for the coherence independent from gene clustering along chromosomes, we did a similar permutation test but filtered out any complexes that have any two members from the genes within 500 kb; >99.5% of the longest length of synteny blocks [55, 56].

For the GO analysis, we used a Cytoscape plugin, BiNGO 2.44 [106]. A hypergeometric test was used to test for significant enrichment of GO terms, and *P*-values were corrected with the Holm-Bonferroni method [107]. Gene lists used as inputs for GO analysis of *S2-DRSC* and *Kc167* cell copy number are in Additional file 4.

### Karyograms

Cells were treated with 1 mM colchicine for 2 hours to disrupt the mitotic spindle. After phosphate-buffered saline washing, we added hypotonic solution (0.5% sodium citrate) by gently dropping (5 ml into a 15 ml tube) and incubated for 10 minutes at room temperature. We centrifuged the cells to remove supernatant, then fixed cells by adding 3:1 (v:v) ice-cold mix of methanol and acetic acid (5 ml) drop-wise. The step was repeated. The supernatant was discarded and the cells were resuspended in 100 μl of fixative and 10 μl was spread and air-dried on a microscope slide. DAPI (1.5 mg/ml) in Vectashield (Vector Laboratories, Burlingame, CA, USA) was used for staining. Chromosome preparations were analyzed using a Zeiss Axioplan fluorescence microscope (Carl Zeiss Microscopy, Oberkochen, Germany) equipped with a CCD camera (CoolSnap HQ, Photometrics, Tucson, AZ, USA). We used Adobe Photoshop to align the karyograms. Detailed interpretation of mitotic spreads is provided in Additional files 1 and 2.

### Data access

All sequencing data described in this manuscript can be found in the Gene Expression Omnibus (GEO) and the SRA. DNA-Seq data for *Cl.8* (#2), *BG3-c2*, *Kc167*, and *S2-DRSC* are in the GEO under accessions GSM697064-5, GSM498672-3, GSM498670-1, and GSM498668-9. Data for the other cell lines as well as Oregon R results used in this study are in the SRA under accessions SRA052953 (SRR497712-8, SRR497720-2, SRR497724-30). *S2-DRSC* (#1) [31] is archived in the GEO under accession GSE16344. The modENCODE transcriptome group produced RNA-Seq data, and results are available in SRA008380 (SRR015074, SRR015076, SRR015078, SRR015080, SRR015082, SRR015084, SRR015086, SRR015088, SRR015090, SRR015092, SRR015094, SRR015096, SRR015098, SRR015100, SRR015102, SRR015104, SRR015106, SRR015108, SRR015110, SRR015112) and SRA009364 (SRR070266, SRR070271-4, SRR070277, SRR070286, SRR07028-9, SRR070291, SRR111868-9, SRR111871, SRR111876-7, SRR189833-5). Copy number calling of the cell lines are provided in Additional files 3 and 4.

ChIP-chip results [88] are in the modENCODE DCC under submission IDs: 201, 274-80, 282-5, 288-99, 301-13, 316-31, 921-2, 924-8, 930, 937-8, 940-67, 2650-1, 2653-5, 2658-60, 2666-74, 2984, 2986-8, 2991, 2994, 2996, 2998-3000, 3002-5, 3007, 3009, 3011, 3013-4, 3016-7, 3019-20, 3026-7, 3029-32, 3035-50, 3052, 3054-8, 3060-2, 3064, 3170, 3279-83, 3286-9, 3291, 3293-6, 3299-304, 3675-6, 3700, 3708, 3710, 3744-5, 3748-53, 3755, 3757-8, 3760-3, 3765, 3768-70, 3777, 3783-92, 3797, 3800, 3803-4, 3894, 3897, 3899, 3941-3, 3945, 3948-50, 4126-7, 4176, 4179, 4182-3, 4185, 4187-8, and 4197.

## Electronic supplementary material

Additional file 1: **Karyograms of all cell lines used in this study.** (PDF 61 KB)

Additional file 2: **A summary of the number of chromosomes and whole chromosome copy number changes from the karyograms.** (PDF 4 MB)

Additional file 3: **Genome-wide copy number in cell lines and copy number breakpoints (1 kb level).** (ZIP 6 MB)

Additional file 4: **Genome-wide copy number and expression data (gene level).** (XLSX 7 MB)

## Abbreviations

bp: base pair
ChIP: chromatin immunoprecipitation
DCC: modENCODE Data Coordination Center
DNA-Seq: DNA sequencing
DPIM: Drosophila Protein interaction Map
DRSC: Drosophila RNAi Screening Center
FDR: false discovery rate
FPKM: fragments per kilobase per million reads
GEO: Gene Expression Omnibus
GO: Gene Ontology
IAP: inhibitor of apoptosis protein
miRNA: microRNA
MSL: male-specific lethal
PDGF: platelet-derived growth factor
PSI: proportion spliced in
RNA-Seq: RNA sequencing
SRA: NCBI Short Read Archive
VEGF: vascular endothelial growth factor
X: A: X chromosomes to autosomes ratio.
