# Correction to: DNA copy number evolution in *Drosophila* cell lines

**DOI:** 10.1186/s13059-019-1668-5

**Published:** 2019-03-11

**Authors:** Hangnoh Lee, C. Joel McManus, Dong-Yeon Cho, Matthew Eaton, Fioranna Renda, Maria Patrizia Somma, Lucy Cherbas, Gemma May, Sara Powell, Dayu Zhang, Lijun Zhan, Alissa Resch, Justen Andrews, Susan E. Celniker, Peter Cherbas, Teresa M. Przytycka, Maurizio Gatti, Brian Oliver, Brenton Graveley, David MacAlpine

**Affiliations:** 1 National Institute of Diabetes, Digestive, and Kidney Diseases, National Institutes of Health, 50 South Drive, Bethesda, MD 20892 USA; 2 Department of Genetics and Developmental Biology, Institute for Systems Genomics, University of Connecticut Health Center, 400 Farmington Avenue, Farmington, CT 06030 USA; 3 Computational Biology Branch, National Center for Biotechnology Information, National Library of Medicine, National Institutes of Health, 8600 Rockville Pike, Bethesda, MD 20892 USA; 4 Department of Pharmacology and Cancer Biology, Levine Science Research Center, Duke University Medical Center, 308 Research Drive, Durham, NC 27708 USA; 5 Istituto di Biologia e Patologia Molecolari (IBPM) del CNR and Dipartimento di Biologia e Biotecnologie, Sapienza, Università di Roma, 5 Aldo Moro Piazzale, 00185 Rome, Italy; 6 Department of Biology, Indiana University, 1001 East 3rd Street, Bloomington, IN 47405 USA; 7 Department of Genome Dynamics, Lawrence Berkeley National Laboratory, 1 Cyclotron Road, Berkeley, CA 94720 USA; 8 Present addresses: Department of Biological Sciences, Carnegie Mellon University, 4400 Fifth Avenue, Pittsburgh, PA 15213 USA; 9 School of Agricultural and Food Science, Zhejiang A and F University, 88 Huan Cheng Bei Road, Lin’an, Zhejiang, 311300 China

**Correction to: Genome Biol (2014) 15:R70**

**http://genomebiology.com/2014/15/8/R70**

Following publication of the original article [1], the authors reported the following errors:

1. In Fig. 3a, both *Drosophila* D20-c2 and D20-c5 cells are shown as D20-c3. The top should be D20-c2 and the bottom should be D20-c5. The updated Fig. 3 is shown below.
2. Labelling of the cell lines in Additional file 3 was incorrect. The updated Additional file 3 is supplied in this correction.

**Fig. 3:**
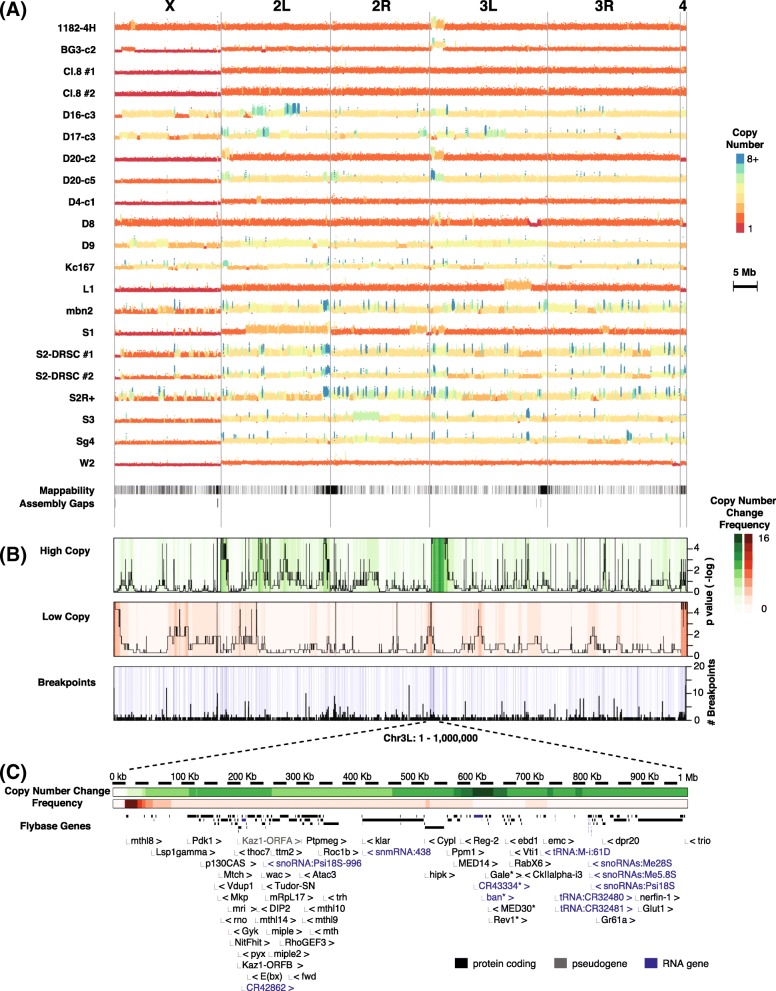
DNA copy numbers. **a** Plots of mapped DNA read density along the genome. Deduced copy number is indicated by color (see key). **b** Heatmaps display how many cell lines have increased (green) or decreased (red) copy number. Black lines in the first two rows show significance. Blue lines indicate breakpoints. Black in the bottom row shows the number of breakpoints shared by the 19 cell lines. **c** A zoomed-in map of the sub-telomeric region (1 Mb) of chromosome 3 L. Asterisks: genes within the highly duplicated regions. Genes with little or no functional information (‘CG’ names) were omitted for brevity

## Additional file

Additional file 3: Genome-wide copy number in cell lines and copy number breakpoints. (XLSX 21852 kb)
