# Supplementary material for: DNA copy number evolution in Drosophila cell lines
Source: Genome Biol. 2014 Aug 28;15(8):R70. doi: 10.1186/gb-2014-15-8-r70 (PMC4289277; doi:10.1186/gb-2014-15-8-r70)
Supplement: Supplementary file 1 — Additional file 1: Karyograms of all cell lines used in this study. (PDF 61 KB) [file 13059_2013_3375_MOESM1_ESM.pdf]

Supplementary Table S1. A summary of the number of chromosomes from the karyograms

Cell line	# of cells scored	# of karyo grams	X:A ratio	Number of chromosomes (%)													
				6	7	8	9	10	11	12	13	14	15	16	17	18	>20
1182-4H	93	8	0.98		19.4	64.5	3.2				3.2	9.7					
BG3-c2	164	22	0.62	7.9	8.5	15.3	9.8	6.7	1.8	4.9	6.1	6.7	13.5	9.1	1.2	8.5	
Cl.8	111	7	0.48	10.8	27.1	29.7	8.1	2.7	5.4		10.8		5.4				
D16-c3	150	10	0.88	25.3	16.0	9.3	10.7	5.3	2.7	6.7	10.7	8.0					5.3
D17-c3	115	18	0.98	5.2	8.7	74.8								11.3			
D20-c5	102	8	0.47					5.9	19.6	23.5	23.5	13.7	2.0				11.8
D4-c1	182	15	0.73	19.2	30.9	22.1	2.2		3.8	2.7	8.2	5.5	1.1			0.5	3.8
D8	81	16	0.98		18.5	68.0	8.6	1.2	1.2	2.5							
D9	135	16	n.d.			1.5	5.9	34.1	14.8	25.2	1.5	2.2	0.7				14.1
Kc167	157	12	0.99						3.8	5.7	8.9	7.6	16.6	21.0	5.1	10.2	21.0
L1	100	6	0.50	94.0	6.0												
S1	119	12	0.51			16.8	57.1	26.1									
S2-DRSC	115	10	0.48									7.0	17.4	20.0	16.5	7.8	31.3
S2R+	145	14	0.58					15.9	12.4	24.8	11.7	9.7	6.9	4.1		6.9	7.6
S3	165	10	0.58			3.0	1.2	12.1	21.2	27.3	12.1	6.1	4.8				12.1
Sg4	112	13	0.50				2.7	18.7	17.0	17.9	8.8	2.7	4.5	3.6		4.5	19.6

Cell line	# of cells scored	# of karyo grams	X:A ratio	Number of chromosomes (%)															
				16	17	18	19	20	21	22	23	24	25	26	27	28	30	>30	>40
mbn2	102	16	0.54	6.9	4.9	5.9	4.9	11.8	2.9	12.7	8.8	10.8	5.9	6.9	2.9	3.9	3.9	4.9	2.0
