# Supplementary material for: DNA copy number evolution in Drosophila cell lines
Source: Genome Biol. 2014 Aug 28;15(8):R70. doi: 10.1186/gb-2014-15-8-r70 (PMC4289277; doi:10.1186/gb-2014-15-8-r70)
Supplement: Supplementary file 2 — Additional file 2: A summary of the number of chromosomes and whole chromosome copy number changes from the karyograms. (PDF 4 MB) [file 13059_2013_3375_MOESM2_ESM.pdf]

<u>Notes for the all karyograms</u>

"**X**, **2**, **3**, or **4**" indicates chromosomes that were either intact or similar to wild-type chromosomes. These included chromosomes with inversions or slightly longer than wild-type forms. "**r**" indicates a deleted or rearranged chromosome that carried the centromeric region of an identifiable autosome. "**t**" indicates a translocation. "**am**" indicates that a centromeric region from a major autosome (2 or 3) that was associated with euchromatic material, "**nd**": not determined.
In the photographic subpanels, lower-case letters matching raw images to karyograms.

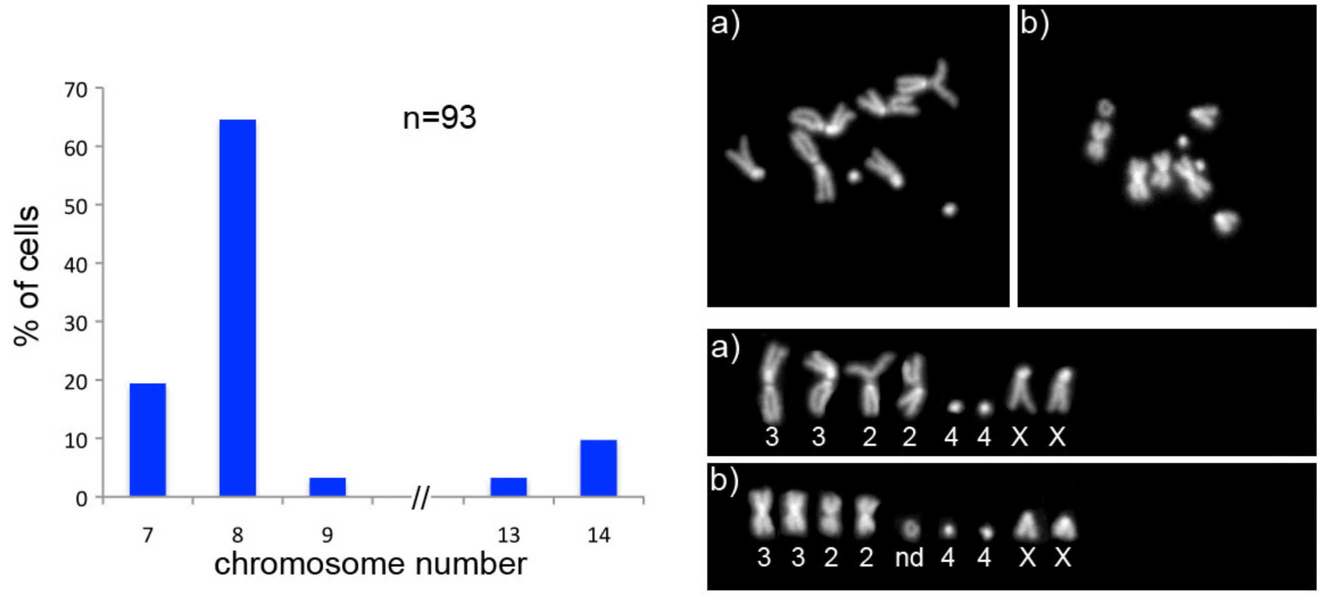

1182-4H **(derived from haploid embryos):**
Left panel. 65% of the metaphases contained 8 chromosomes with a normal diploid karyotype. 19% of the cells displayed seven chromosomes, with three autosomes instead of four. However, a fraction of the latter metaphases could be diploid metaphases that had lost an autosome during the fixation procedure. 13% of the metaphases were hyperploid with ~14 chromosomes (eight autosomes, three X chromosomes and three 4th chromosomes).
Right panel. All chromosomes looked wild-type in morphology except a small chromosome found in a single karyotype (b). This chromosome looked like a small ring and probably carried heterochromatin at both ends.
Sex. In the 30 metaphases analyzed the average X:A ratio of 0.98 (Table S1). The observed karyotype agrees with the sequencing data indicating that this line has a female karyotype and expression pattern.
Note. This line is now diploid.

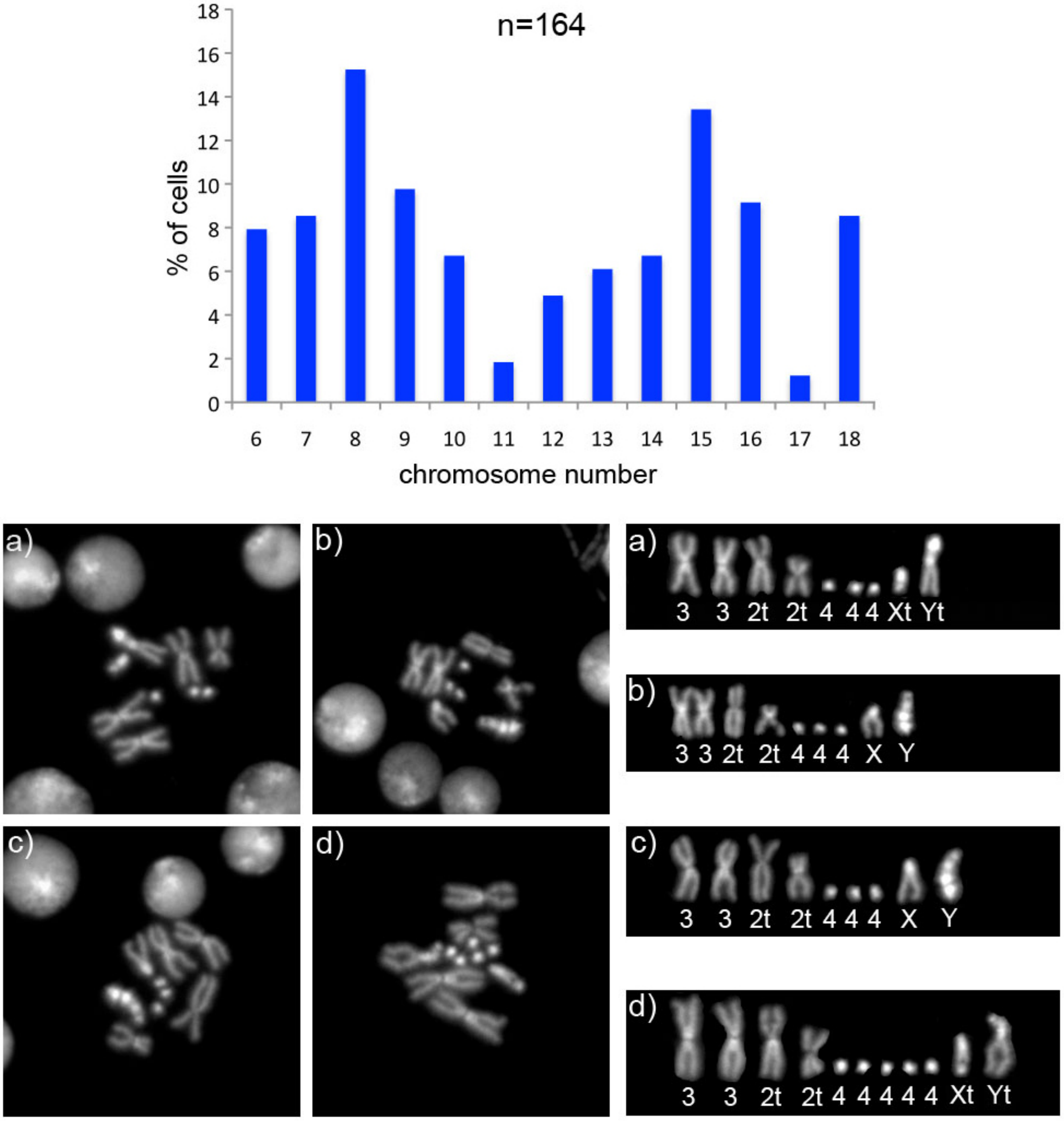

***BG3-c2* (derived from larval central nervous system):**

Top panel. 34% of the metaphases contained 7-9 chromosomes. 32% exhibited 15-18 chromosomes with quasi-tetraploid character.

Bottom panel. In most of the cells, a translocation between 2nd chromosomes (2t) was observed. While some karyograms displayed apparently normal X and Y chromosomes (b, c), others showed the two elements of an X-Y translocation (Xt or Yt) with the entire euchromatic part of XL appended to the long arm of the Y (XL-YL·YS) and part of YL appended to the heterochromatic block of XL (XS·XL-YL).

Alternatively, the XL^{-}YL$^{\cdot}$YS element of this translocation with no XS$^{\cdot}$XL-YS element was also observed (d and a, respectively). In 27% of the cells, there was a short (probably deleted) derivative of the XL^{-}YL$^{\cdot}$YS element accompanied by a regular X, or a canonical (undeleted) XL^{-}YL$^{\cdot}$YS element, or both. The number of 4th chromosomes was highly varied. (1-5 in quasi-diploid cells and 2-8 in quasi-tetraploid cells.)

Sex. In the 22 karyograms analyzed the average X:A ratio was 0.62 (Table S1). However, we also observed that this ratio was ~ 1 in 23% of the examined cells. The observed karyotype agrees with the sequencing data indicating that this line has a male karyotype and expression pattern.

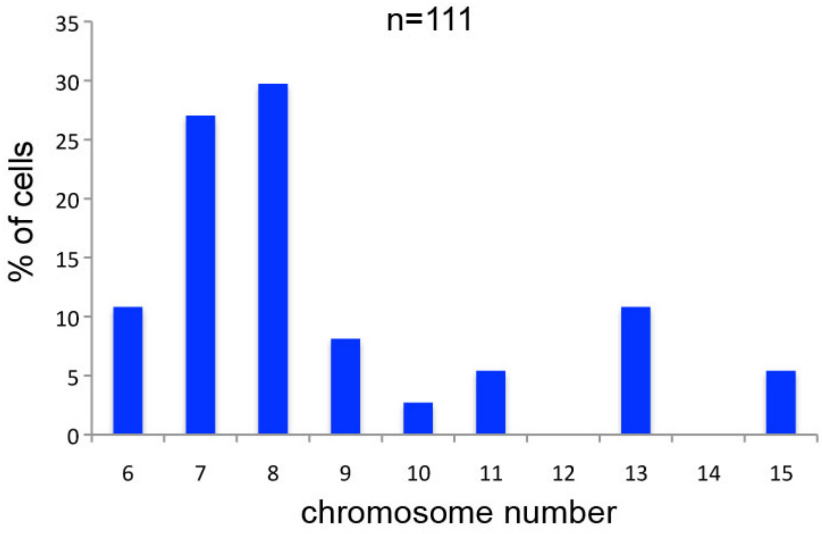

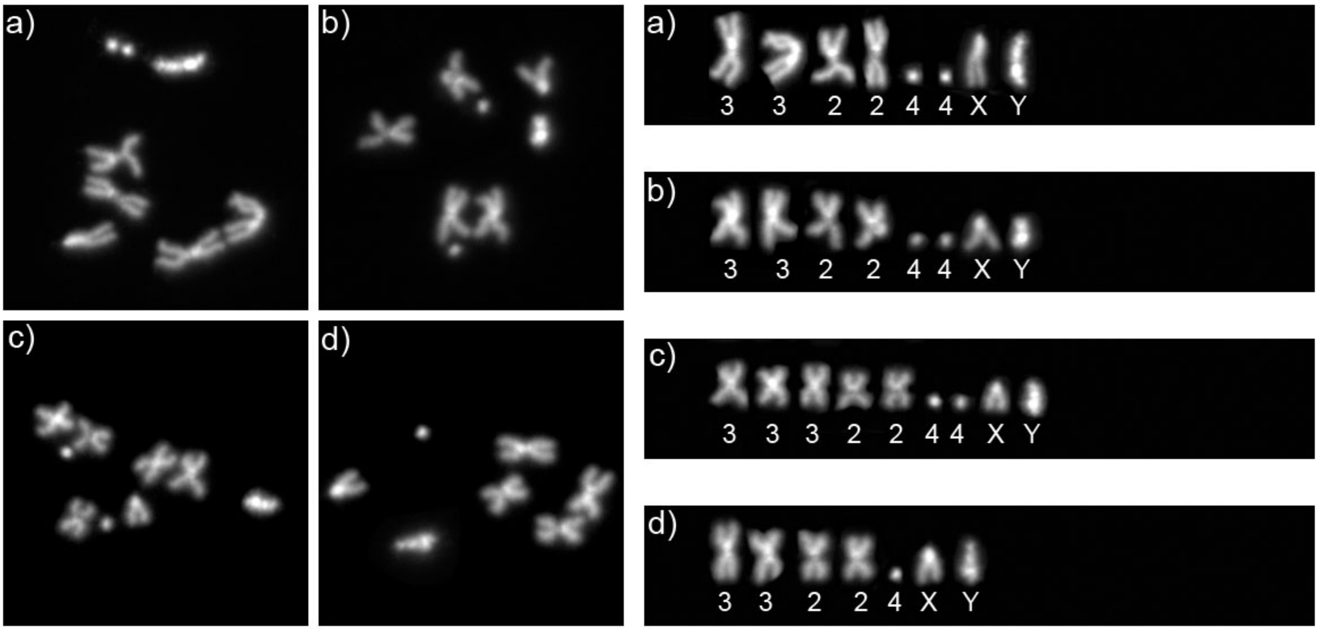

Cl.8 **(derived from larval stage 3 wing disc):**

Top panel. 76% of the metaphases contained 6-9 chromosomes. 16% exhibited 13-15 chromosomes and were probably derived from polyploidization of cells with 6-9 chromosomes.

Bottom panel. Most cells exhibited four metacentric autosomes, one X chromosome, one Y chromosome and no to three 4th chromosomes. The metacentric autosomes, the X chromosome and 4th chromosomes looked wild-type in morphology. The Y chromosome was shorter than the wild-type counterpart and seemed to carry a deletion of the nucleolus organizer region (b, c).

Sex. In the 30 karyograms analyzed the average X:A ratio was 0.48 (Table S1). The observed karyotype agrees with the sequencing data indicating that this line has a male karyotype and expression pattern.

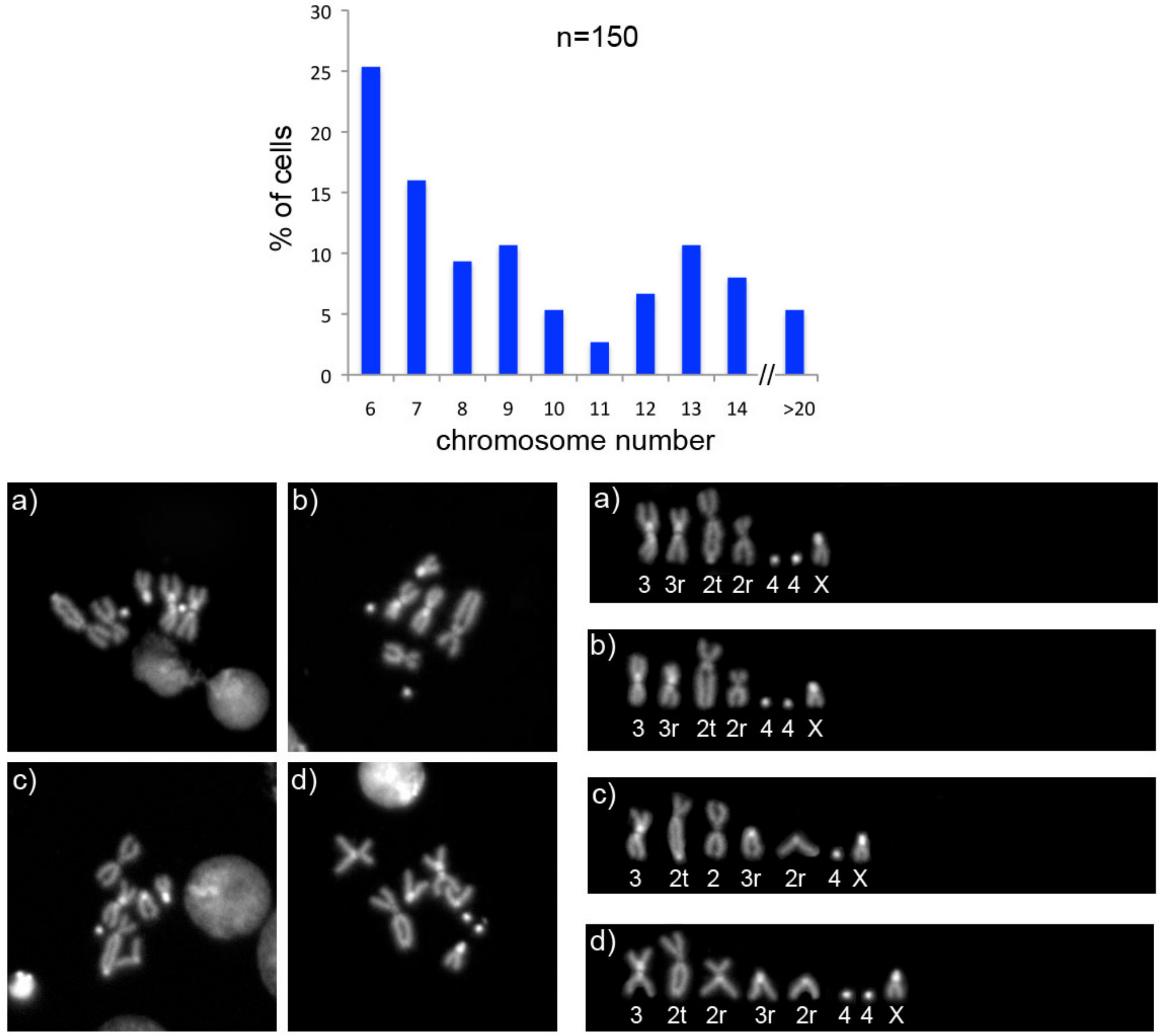

D16-c3 **(derived from larval stage 3 wing disc):**

Top panel. 61% of the metaphases contained 6-9 chromosomes. 25% exhibited 12-14 chromosomes and were probably derived from polyploidization of cells with 6-9 chromosomes.

Bottom panel. All cells exhibit large marker chromosome generated by an X-2 translocation (2t). This chromosome contained an almost entire 2nd chromosome with the left arm conjoined to the left arm of an almost entire X chromosome, which lacked the centromere but retains some pericentromeric heterochromatin. The large X-2 translocation element was accompanied by various combinations of regular or rearranged metacentric autosomes, including a 3rd chromosome carrying a deficiency of part of 3L euchromatin, a 2nd chromosome with an abnormally short arm (it was impossible to assess which

arm was partially deleted), a 3rd chromosome lacking the entire 3R arm (3r), and a 2nd chromosome lacking the entire 2R arm (2r). The X and 4th chromosomes looked wild-type in morphology.

Sex. In the 10 karyograms analyzed the average X:A ratio was 0.88 (Table S1). The observed karyotype agrees with the sequencing data indicating that this line has a female karyotype and expression pattern.

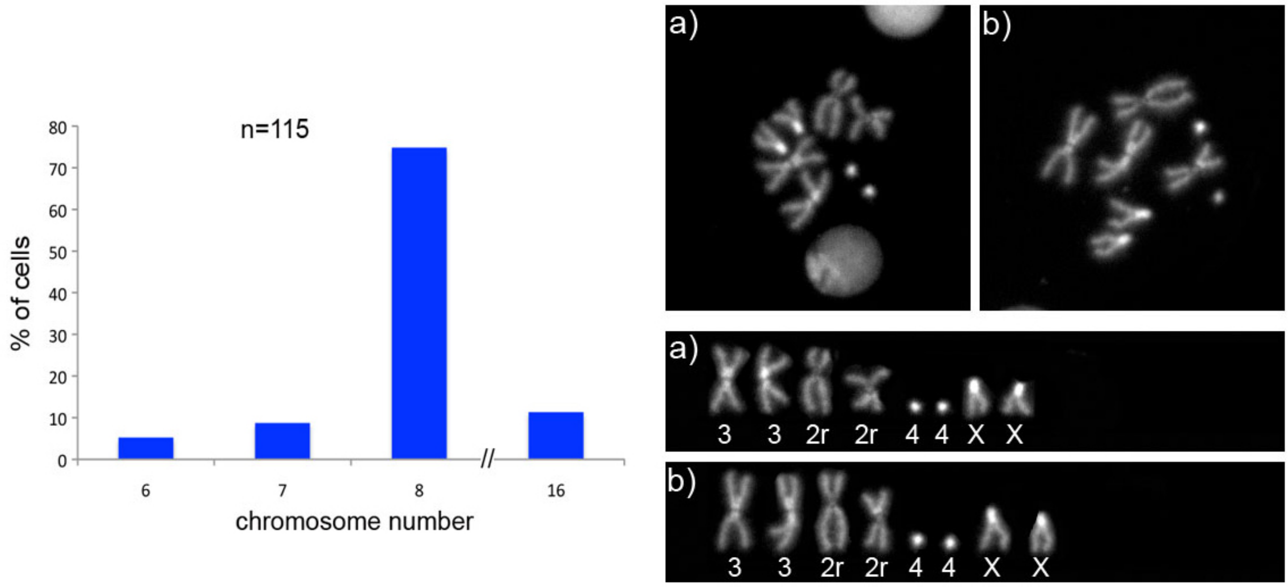

D17-c3 **(derived from haltere disc):**

Left panel. 89% of the metaphases contained 6-8 chromosomes. 11% exhibited 16 chromosomes and were probably derived from polyploidization of cells with 6-9 chromosomes.

Right panel. The most cells exhibited four large metacentric chromosomes. In most cases, one of the 2nd chromosomes had abnormally long 2L arm and the other an abnormally short 2L arm (2r). These rearranged chromosomes were probably the result of a translocation. We also observed two types of X chromosomes displaying a small difference in the length of the euchromatic long arm (b). Karyograms displayed two X chromosomes from the most of cells, as well as two 4th chromosomes.

Sex. In the 18 karyograms analyzed the average X:A ratio was 0.98 (Table S1). The observed karyotype agrees with the sequencing data indicating that this line has a female karyotype and expression pattern.

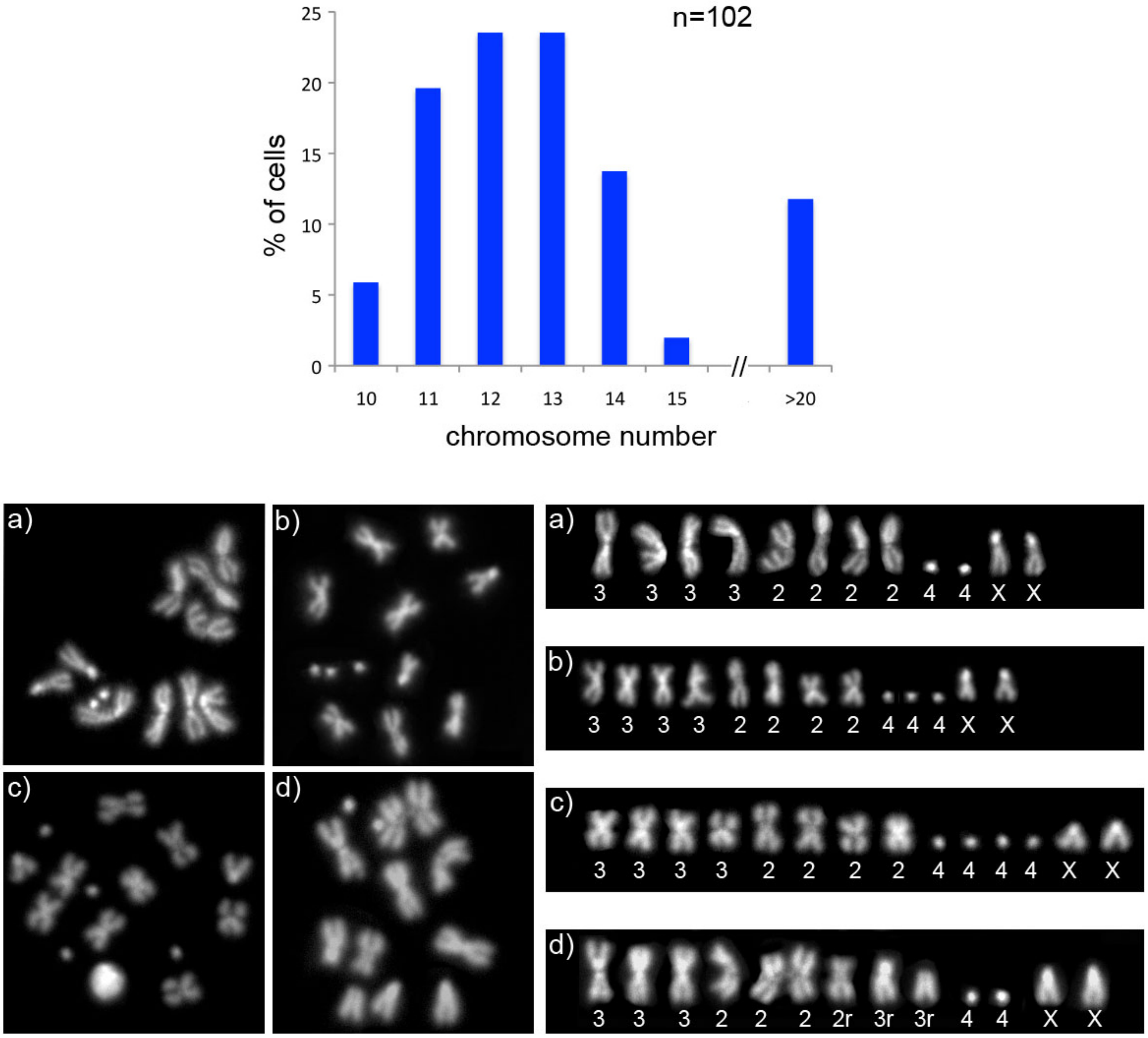

D20-c5 **(derived from antennal disc):**

Top panel. 80% of the metaphases contained 11-14 chromosomes (four 3rd chromosomes four 2nd chromosomes, 2 X chromosomes and a variable number of 4th chromosomes). 12% exhibited more than 20 chromosomes and were probably derived from polyploidization of cells with 11-14 chromosomes.

Bottom panel. In most of the cells, the chromosomes exhibited relatively normal morphology, but the pericentric heterochromatin of one pair of 2nd chromosomes was longer (clear in b) that has been seen in Oregon R. We believe that this peculiar pericentric region reflects a natural polymorphism rather than a rearrangement. In one karyogram, there was a 2nd chromosome with a deletion, and a likely X-3 (or a 4-3 translocation) (d).

Sex. In the 20 karyograms analyzed the average X:A ratio was 0.47 (Table S1). The observed karyotype agrees with the sequencing data indicating that this line has a male karyotype and expression pattern.

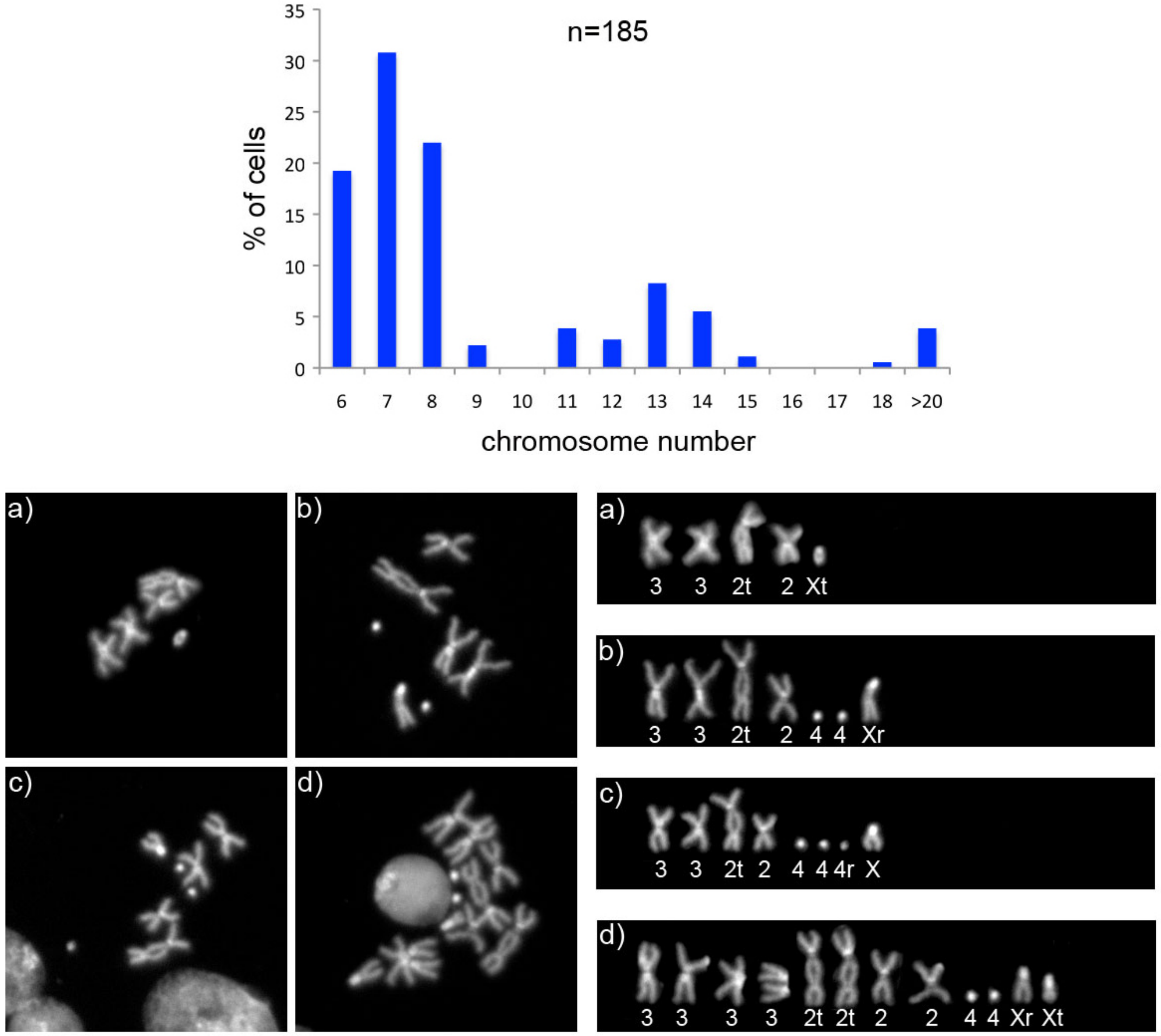

***D4-c1* (derived from larval stage 3 mixed discs):**

Top panel. 72% of the metaphases contained 6-8 chromosomes. 15% exhibited 12-14 chromosomes and were probably derived from polyploidization of cells with 6-8 chromosomes.

Bottom panel. In most cells, we observed a translocation that appeared to involve the 2nd and the X chromosome and attached most of XL to the terminus of 2R (2t). The large and the small element of this translocation were present in 40% and 7% of the cells examined, respectively. The translocation elements were independently distributed among the cells, and metaphases carrying both elements were rare (a). The autosomes not involved in the translocation had normal appearance, while the free X chromosome exhibited a heterochromatic block bigger than that of a wild-type X (Xr) (b, d).
Sex. In the 16 karyograms and 40 metaphases analyzed the average X:A ratio was 0.73 where we considered translocation's largest element was carrying most of XL (Table S1). Specifically, 23 cells displayed a male karyotype and 27 a female karyotype. The sequencing data indicated a male karyotype and expression pattern.
Note. Somatic pairing was maintained in most cells.

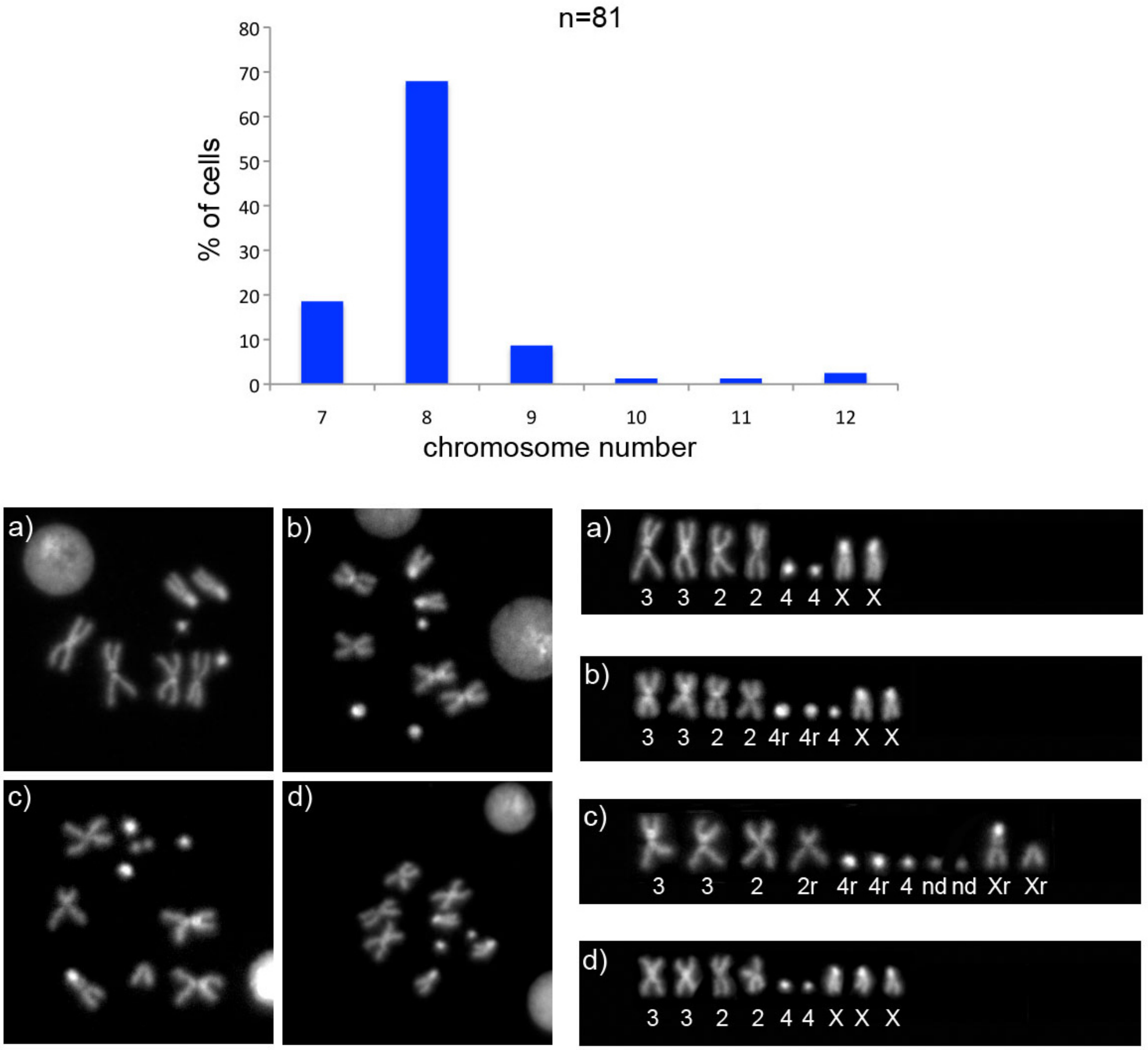

D8 **(derived from larval stage 3 wing disc):**

Top panel. 68% of the metaphases contained 8 chromosomes and exhibited a normal diploid karyotype. 19% exhibited 7 chromosomes. Of the 15 cells with 7 chromosomes, 13 lacked one of the fourth chromosomes and 2 lacked one of the X chromosomes. Most of the hyperploid cells had additional X or 4th chromosomes.

Bottom panel. In most cells, the autosomes and the X chromosomes looked wild-type in morphology but the 4th chromosomes were abnormal (4r in b, c). In one karyogram there was a complex rearrangement involving the X chromosome and possibly the 2nd chromosome (c).

Sex. In the 16 karyograms analyzed the average X:A ratio was 0.98 (Table S1). The observed karyotype agrees with the sequencing data indicating that this line has a female karyotype and expression pattern.

Note. The homologous chromosomes of this line were somatically paired as in wild-type brain cells.

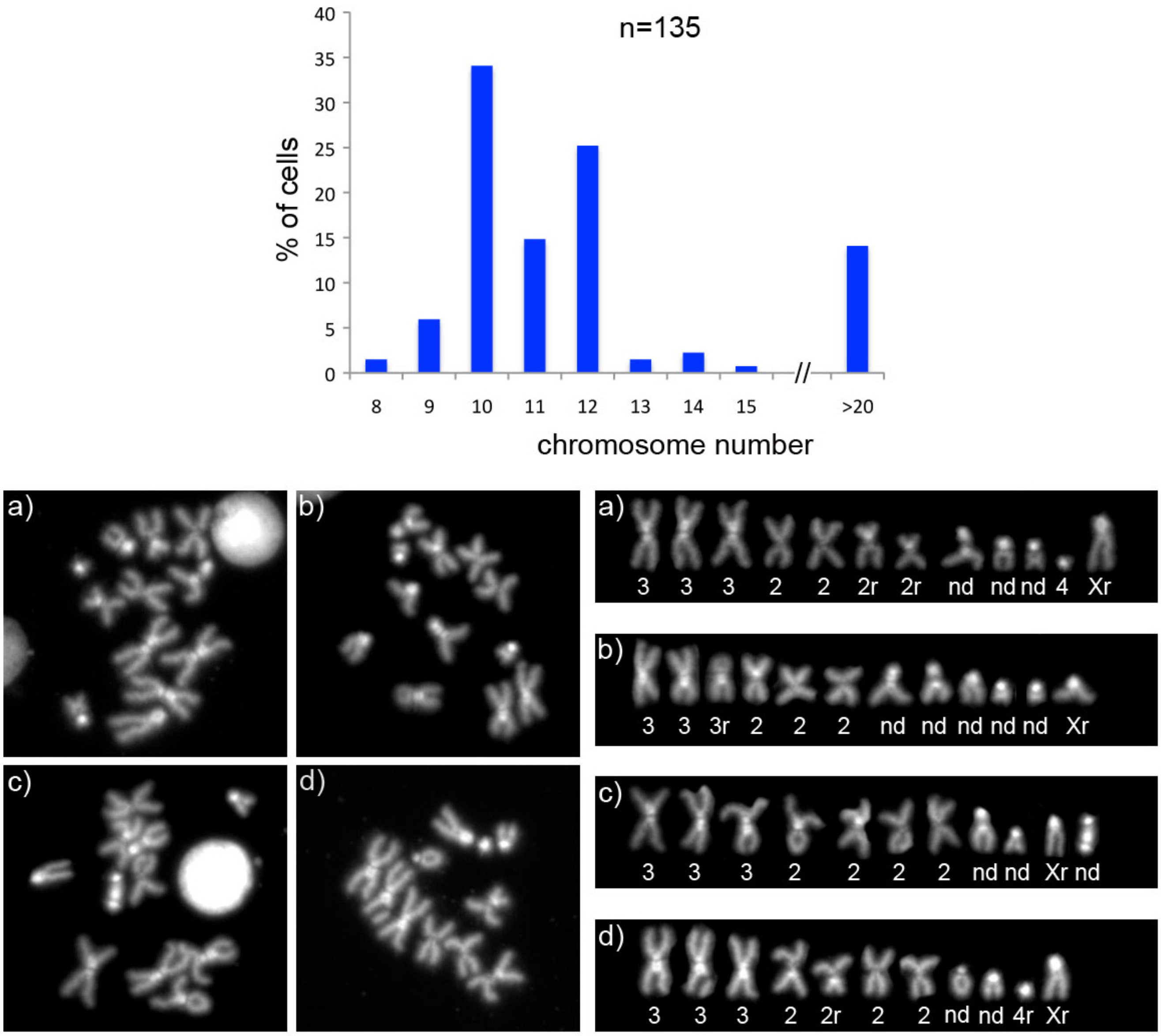

***D9* (derived from larval stage 3 wing disc):**

Top panel. 75% of the metaphases contained 10-12 chromosomes. 14% exhibited more than 20 chromosomes and were probably derived from polyploidization of cells with 10-12 chromosomes.

Bottom panel. Most metaphases exhibited 7-8 major autosomes including normal 2nd and 3rd chromosomes and abnormal derivatives of these autosomes. These derivatives included a 3rd chromosome carrying a deletion of part of 3R, a 3rd chromosome carrying a deletion of an entire arm, a 2nd chromosome carrying a deletion of an entire arm, and two additional rearranged and shorter derivatives of either the 2nd or the 3rd or the chromosome. All cells contained an X chromosome longer

than its wild-type counterpart and variable number of potential X derivatives including a relatively large acrocentric chromosome bearing two blocks of fluorescent pericentric heterochromatin and two smaller chromosomes that could be either X chromosomes carrying a deletion of part their euchromatin, or elements of translocations involving the 4th chromosome and either the X or the major autosomes (c, d). The second alternative was supported by the fact that the average number of free 4th chromosomes was quite low (0.7/cell). We observed several additional rearranged chromosomes of uncertain origin including an element that looked like a partially deleted Y chromosome (nd).
Sex. Given the difficulty in recognizing the X chromosomes we could not determine the X:A ratio. The sequencing data indicated X:A ratio of 0.86, but with male-like gene expression pattern.

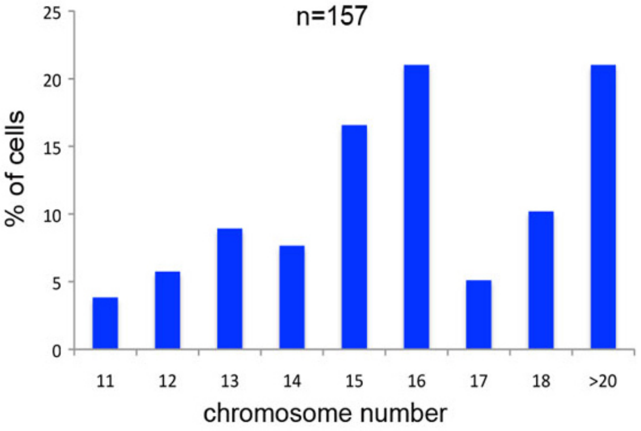

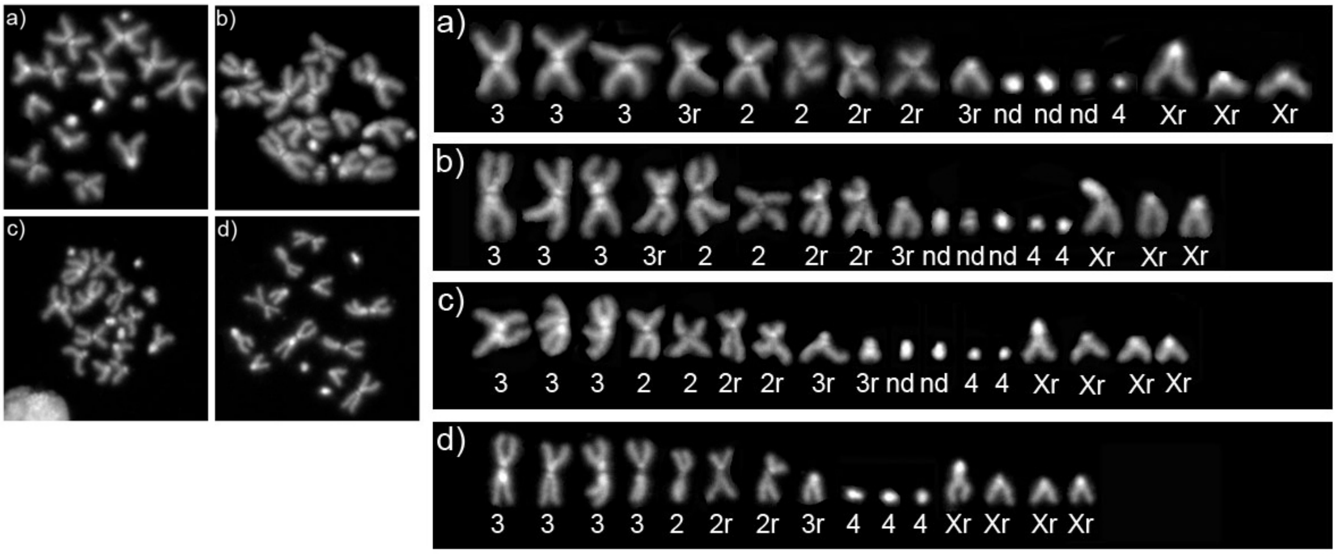

***Kc167* (derived from embryo):**

Top panel. 54% of the metaphases contained 13-16 chromosomes with quasi-tetraploid characters. 21% exhibited more than 20 chromosomes.

Bottom panel. In most cells, they exhibited four 3rd chromosomes and four 2nd chromosomes but these chromosomes were often rearranged (2r and 3r). In addition they carried small derivatives of the major autosomes (a, b). We observed 3rd chromosomes with a deficiency of part of 3L, 3rd chromosomes with deficiencies of both 3L and 3R, a 3rd chromosome lacking the entire 3L arm, and 2nd chromosomes carrying a deficiency of 2L (3r and 2r). The X chromosomes varied in number (from 3 to 4) and morphology. One of the X chromosomes exhibited a fluorescent heterochromatic block bigger than that of a wild-type X chromosome, while some X chromosomes had heterochromatic blocks smaller than

those seen in wild-type (Xr). Some X chromosomes were deleted and contained the proximal heterochromatic block. The dot-like chromosomes varied in morphology and number, ranging from 2 to 5. Some of these small and brightly fluorescent chromosomes were bigger than a normal 4th chromosome (a-c). They could result from rearrangements involving two 4th chromosomes or could be grossly deleted X chromosomes that carry just the most florescent part of X heterochromatin.

Sex. In the 12 karyograms analyzed the average X:A ratio was 0.99 (Table S1). The observed karyotype agrees with the sequencing data indicating that this line has a female karyotype and expression pattern.

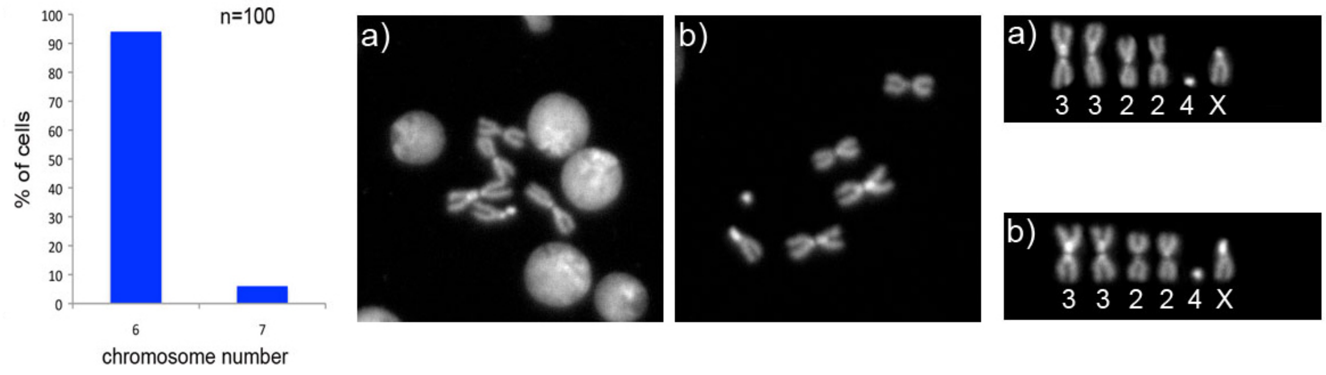

***L1* (derived from larval stage 3 leg disc):**

Left panel. 94% of the metaphases contained 6 (mostly, two 2nd chromosomes, two 3rd chromosomes, a single X chromosome and single 4th chromosome). 6% exhibited 7 chromosomes.

Right panel. In most cells, all chromosomes looked wild-type in morphology. We observed cases of two 4th chromosomes, or a single X chromosome and no 4th chromosomes (from two, or one out of 36 mataphases, respectively)

Sex. In the 36 metaphases analyzed the average X:A ratio was 0.95 (Table S1). The observed karyotype agrees with the sequencing data indicating that this line has a male karyotype and expression pattern.

Note. The homologous chromosomes of this line were somatically paired as in wild-type brain cells.

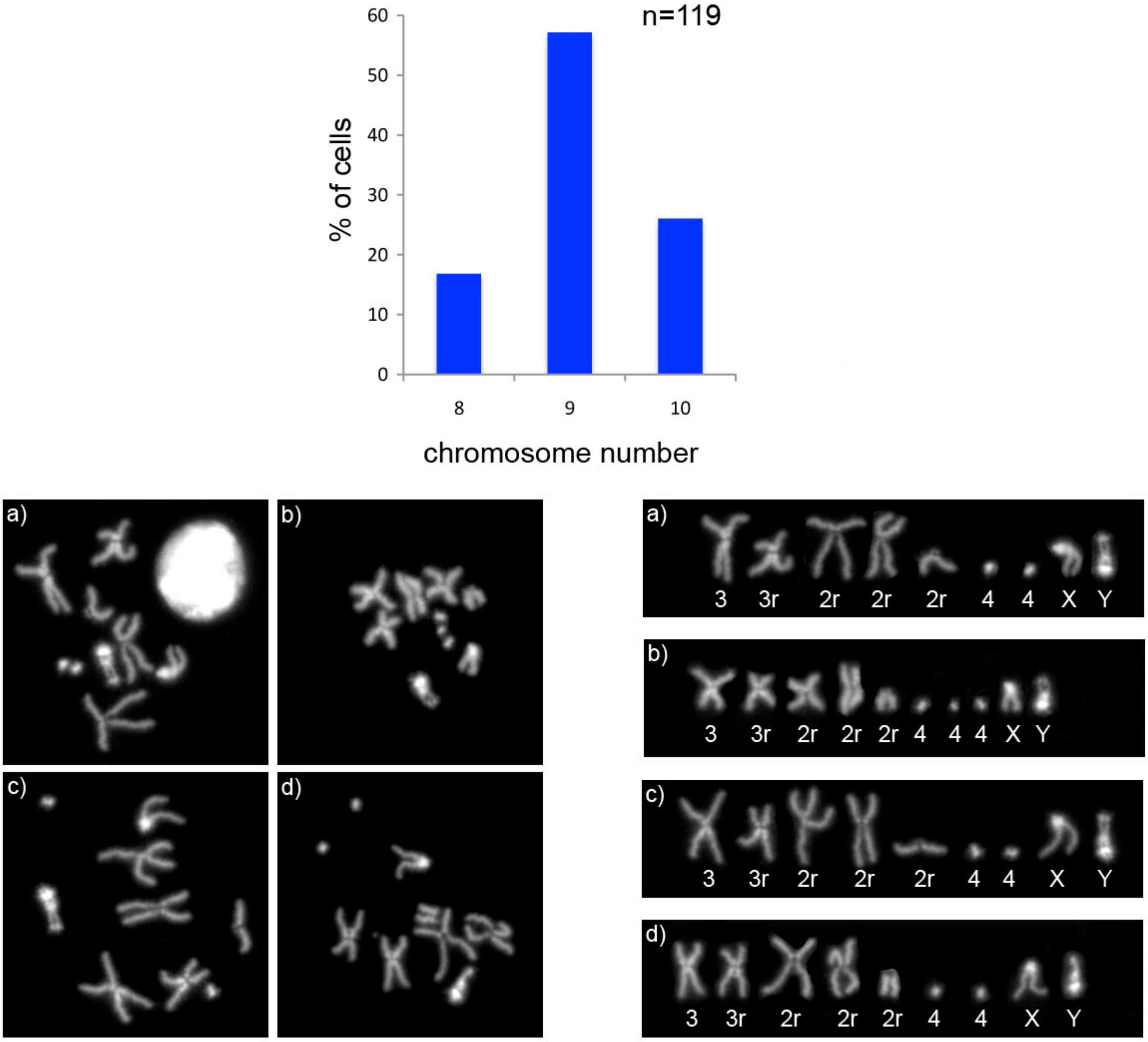

***S1* (derived from embryo):**

Top panel. 100% of the metaphases contained 8-10 chromosomes. 57% of them had 9 chromosomes.

Bottom panel. In most cells, we observed a pair of irregular 2nd chromosomes (both carrying an abnormally long 2R arm), a pair of 3rd chromosomes (one regular and the other carrying a deficiency that removed part of 3R euchromatin), an acrocentric chromosome that looked like a 2L arm (2r) based on heterochromatin banding pattern. This observation was consistent with DNA-Seq data that indicated an excess of 2L sequences compared to 2R, 3L and 3R sequences. A single regular X chromosome, a Y chromosome and two 4th chromosomes were found from the most karyograms, yet the number of dot chromosome was variable ranging from 2 to 3.

Sex. In the 12 karyograms analyzed the average X:A ratio was 0.51 (Table S1). The observed karyotype agrees with the sequencing data indicating that this line has a male karyotype and expression pattern.

Note. In several metaphases the Y chromosome had an unusual appearance, as it exhibited sister chromatid separation in the short arm region that included the ribosomal genes and, less frequently, in the long arm region that contains the *cry*/*su(Ste)* sequences. This separation was never seen in the Y chromosome of larval neuroblasts. However, in wild-type primary spermatocytes, where the Y is functioning, the Y sister chromatids are well separated (like those of the X chromosome and the autosomes). Thus, it is possible that the Y regions that exhibited sister chromatid separation in *S1* cells are transcriptionally active.

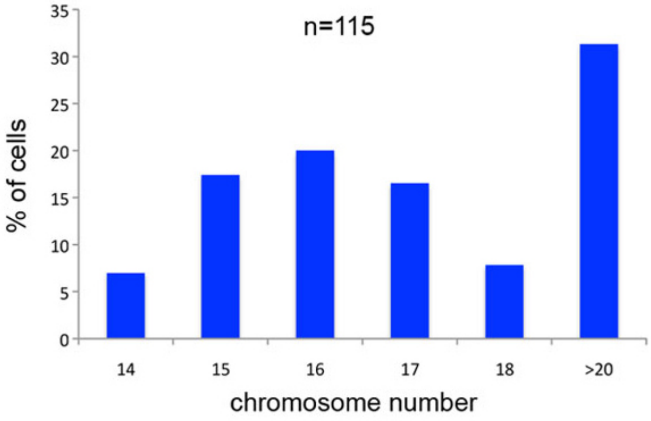

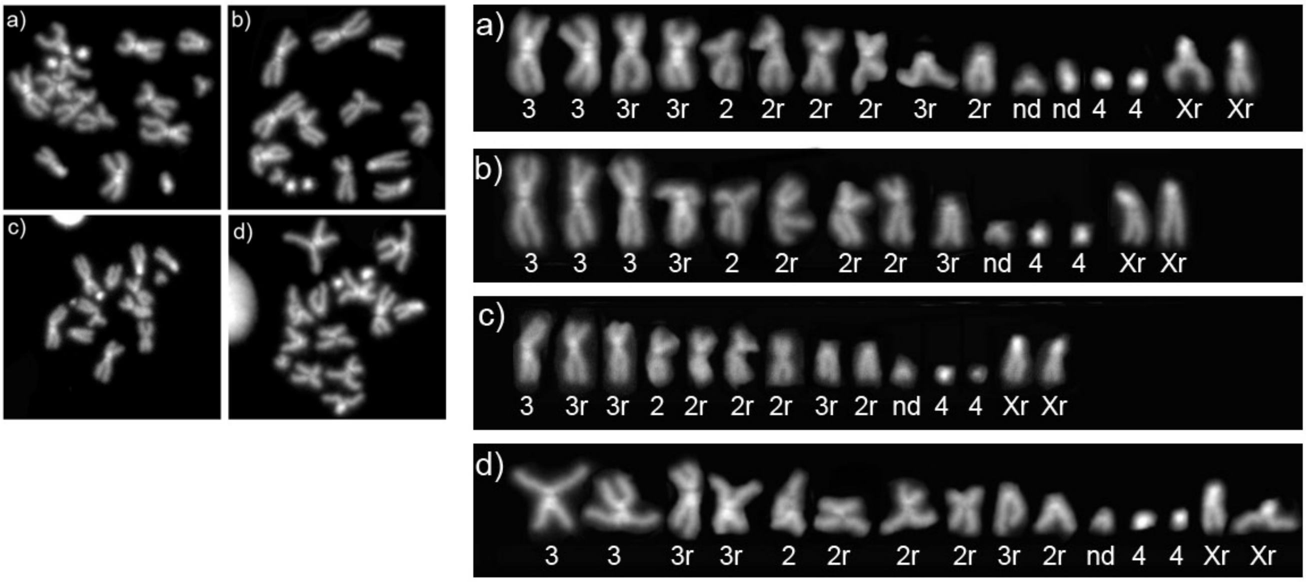

***S2-DRSC* (derived from embryo):**

Top panel. 69% of the metaphases contained 14-18 chromosomes with quasi-tetraploid characters. 31% exhibited more than 20 chromosomes and were probably derived from polyploidization of cells with 14-18 chromosomes.

Bottom panel. Many of the cells exhibited four 3rd chromosomes, some of which carried a pericentric inversion, and four 2nd chromosomes, some of which carried a deficiency of part of 2L euchromatin. We observed one or two acrocentric chromosomes that looked like 2L and 3R arms based on heterochromatin banding pattern. One to three small acrocentric chromosomes that were probably derived from metacentric autosomes were observed. Karyograms exhibited two X chromosomes whose heterochromatic blocks were bigger than wild-type X chromosomes (Xr). Some cells displayed a short

chromosome that was likely to be the heterochromatic portion of an X chromosome (a). The number of 4th chromosome varied from 1 to 3.

Sex. In the 10 karyograms analyzed the average X:A ratio was 0.48 (Table S1). The observed karyotype agrees with the sequencing data indicating that this line has a male karyotype and expression pattern.

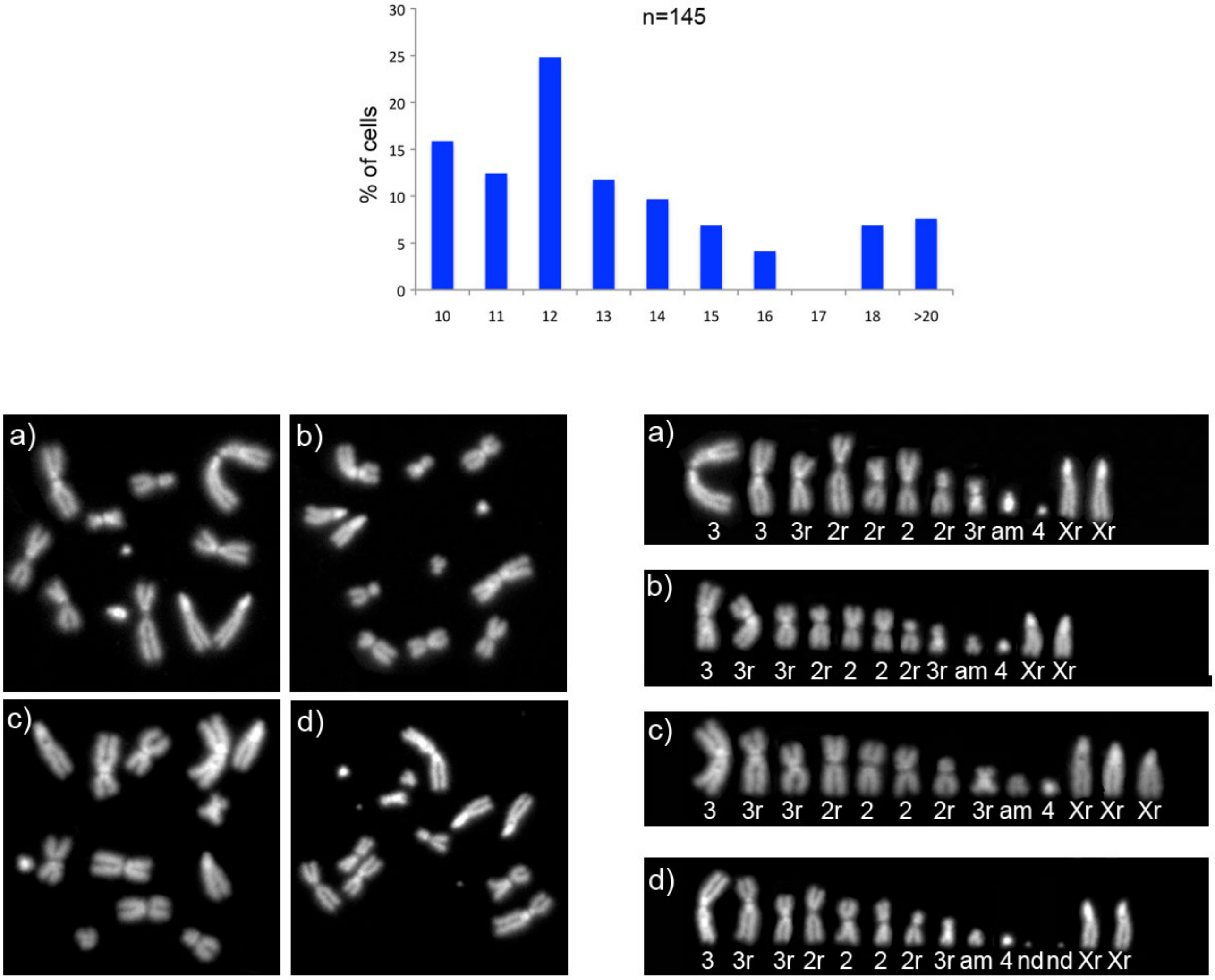

S2R+ (derived from embryo):

Top panel. 70% of the metaphases contained 10-14 chromosomes. 8% exhibited more than 20 chromosomes and were probably derived from polyploidization of cells with 10-14 chromosomes.

Bottom panel. In most of the cells, we observed highly rearranged chromosomes. They included a 3rd chromosome slightly bigger than a normal 3rd with a small insertion of fluorescent heterochromatin in the middle of 3R, a 3rd chromosome carrying deletion of part of the 3R arm, a 3rd chromosome carrying

deletions in both arms, a 2nd chromosome with an abnormally long arm (either a pericentric inversion or an element of a translocation), a 2nd chromosome with a partially deleted 2L arm, and a small acrocentric chromosome that exhibited a centromeric region similar to those of the metacentric autosomes. Also the X chromosomes were rearranged and their euchromatic arms appeared longer than those of wild-type X chromosomes (Xr). They were probably derived from translocations involving the X and one of the autosomes. The dot chromosomes too were highly variable in morphology, size and numbers (ranging from 1 to 4).

Sex. In the 14 karyograms analyzed the average X:A ratio was 0.58 where we considered the amount of autosomal material and not the number of metacentric autosome elements (Table S1). The observed karyotype agrees with the sequencing data indicating that this line has a male karyotype and expression pattern.

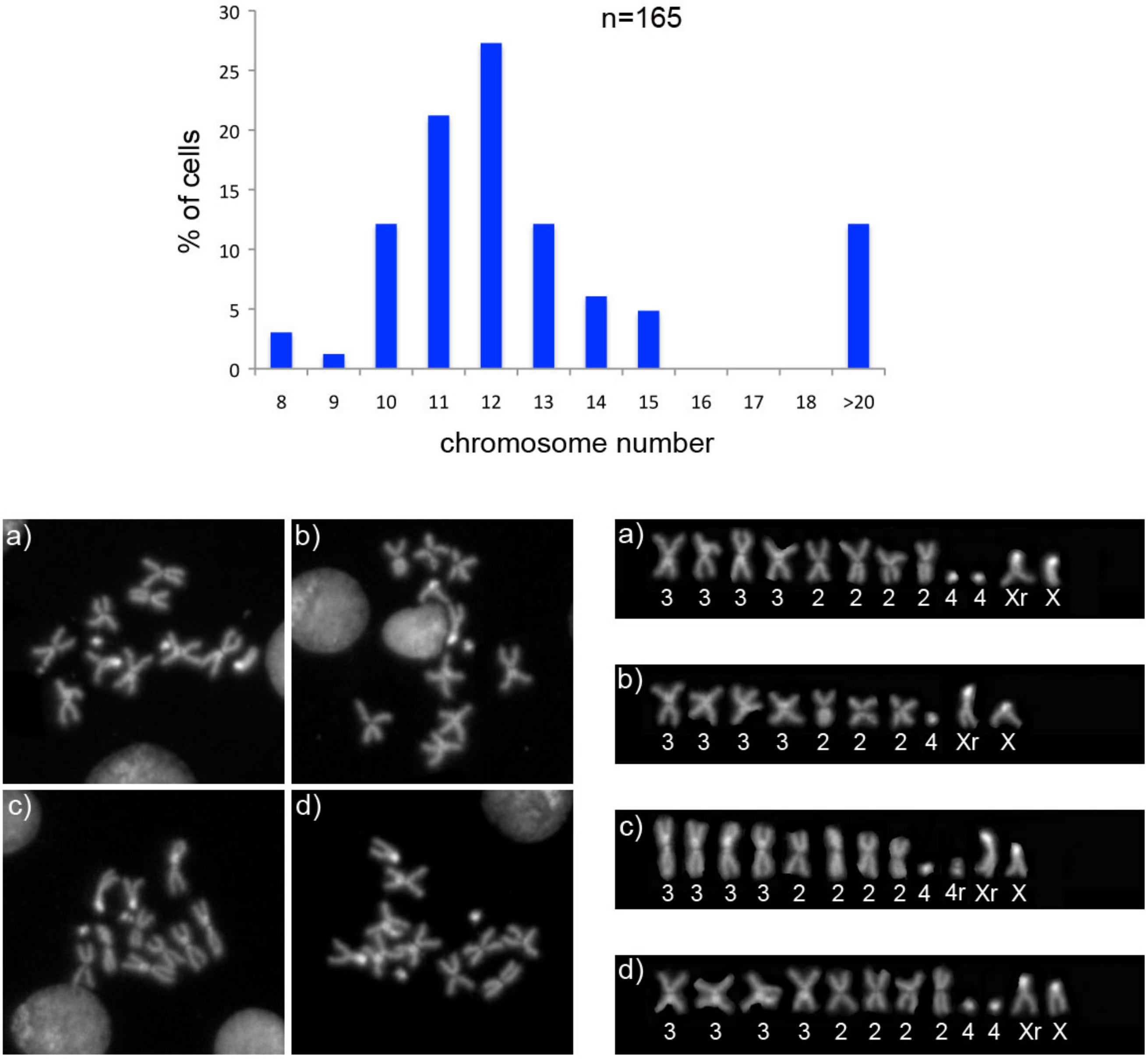

***S3* (derived from embryo):**

Top panel. 83% of the metaphases contained 10-14 chromosomes. 14% exhibited more than 20 chromosomes and were probably derived from polyploidization of cells with 10-14 chromosomes.

Bottom panel. Most cells exhibit 8 morphologically normal metacentric autosomes (four 3rd chromosomes and four 2nd chromosomes), 2 X chromosomes. Thus, these cells generally contained a tetraploid number of autosomes and diploid numbers of X chromosomes. There were two types of X chromosomes. One looked wild-type and the other exhibited a proximal heterochromatic block longer that that seen in wild-type (Xr). The 4th chromosomes were usually normal but with abnormal sizes compare to that of wild-type (4r in c) and the number varied from 1 to 3.

Sex. In the 10 karyograms analyzed the average X:A ratio was 0.58 (Table S1). The observed karyotype agrees with the sequencing data indicating that this line has a male karyotype and expression pattern.

Note. The karyotype of the *S3* cells was extraordinarily similar to that of *Sg4* cells (below). This was also represented in the sequencing data.

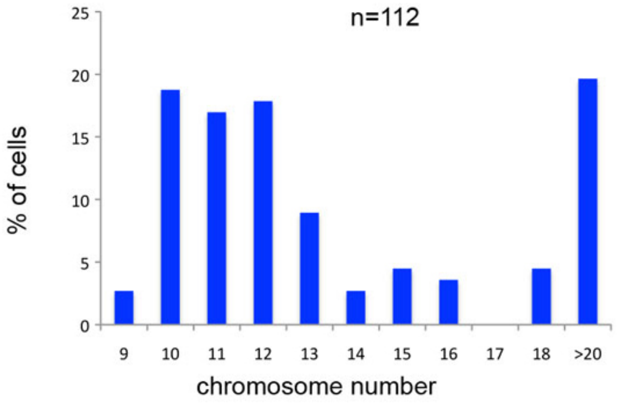

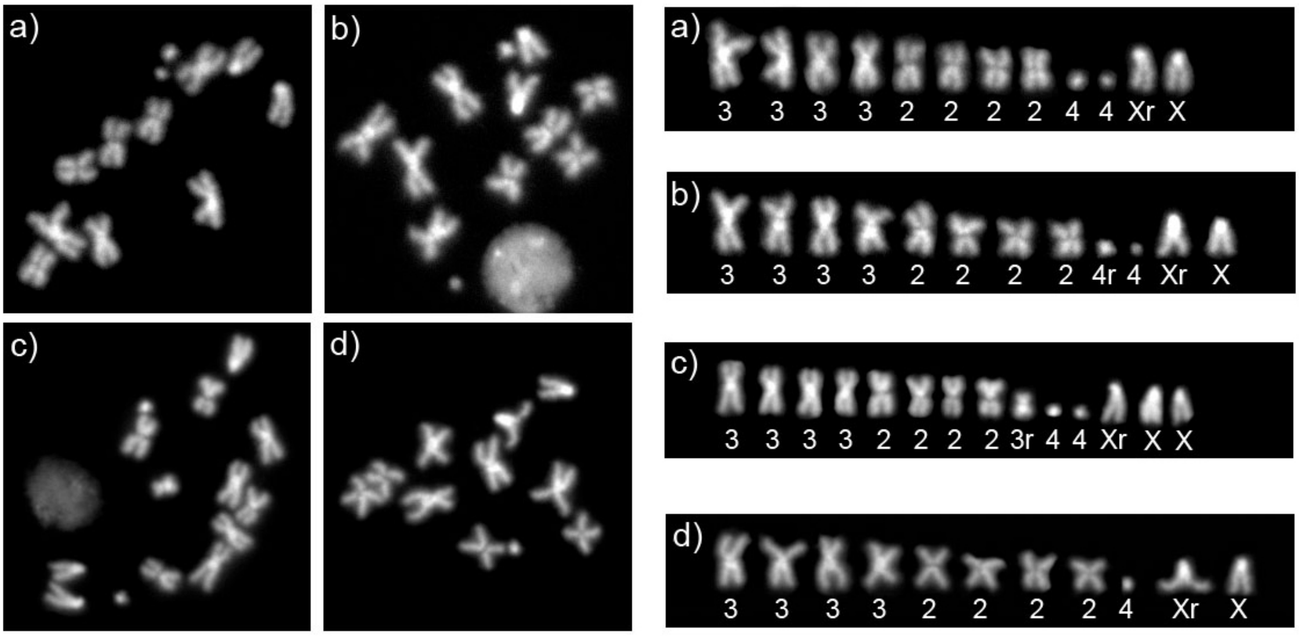

Sg4 **(derived from embryo):**

Top panel. 60% of the metaphases contained 10-13 chromosomes. 22% exhibited more than 20 chromosomes and were probably derived from polyploidization of cells with 10-13 chromosomes.

Bottom panel. Most of the 10-13 chromosome cells displayed relatively normal metacentric autosomes (four 3rd chromosomes and four 2nd chromosomes). They looked wild-type in morphology, but in a few cells, we observed a 3rd chromosome carrying deletions of both arms (c). X chromosomes appeared with

variable numbers ranging from 1 to 3. Thus, the cells of this line generally contained a tetraploid number of autosomes and diploid numbers of X chromosomes. We identified two types of X chromosomes. One looked wild-type and the other exhibited a proximal heterochromatic block longer that that seen in wild-type (Xr). Some of the 4th chromosomes displayed abnormal sizes with varying numbers of 1 to 3.

Sex. In the 13 karyograms analyzed the average X:A ratio was 0.50 (Table S1). The observed karyotype agrees with the sequencing data indicating that this line has a male karyotype and expression pattern.

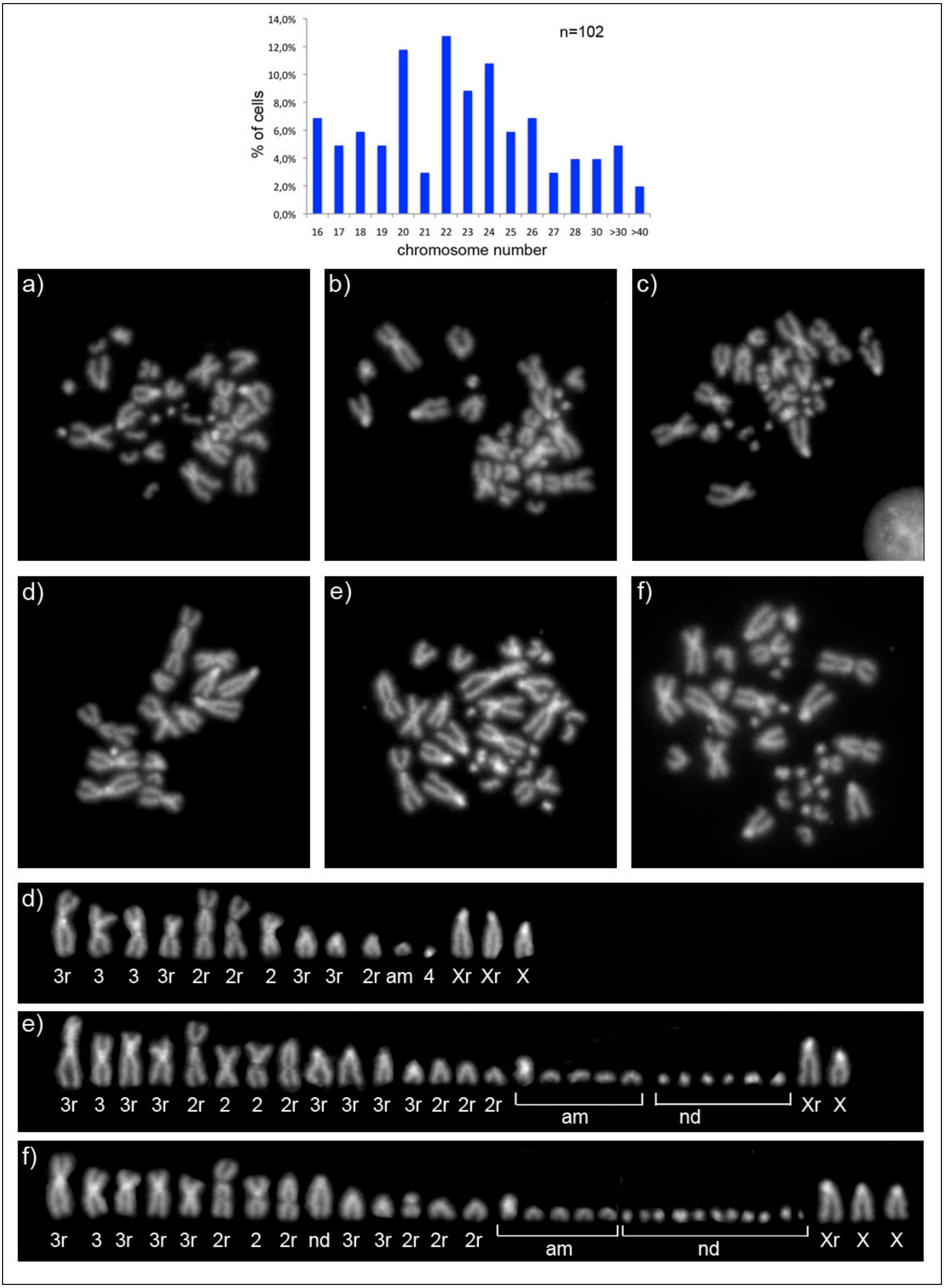
n=102
% of cells
14,0%
12,0%
10,0%
8,0%
6,0%
4,0%
2,0%
0,0%
16 17 18 19 20 21 22 23 24 25 26 27 28 30 >30 >40
chromosome number
a)
b)
c)
d)
e)
f)
d)
3r 3 3 3r 2r 2r 2 3r 3r 2r am 4 Xr Xr X
e)
3r 3 3r 3r 2r 2 2 2r 3r 3r 3r 3r 2r 2r 2r am nd Xr X
f)
3r 3 3r 3r 3r 2r 2 2r nd 3r 3r 2r 2r 2r am nd Xr X X

***mbn2* (derived from hemocytes):**

Top panel. This line was characterized by extremely high karyotypic variability and by the presence of spontaneous chromosome breakage. Chromosome counts were carried out without distinguishing between broken and intact chromosomes. The result showed that most cells were polyploid and contained between 16 and 30 chromosomes.

Bottom panel. A wide variety of chromosome types were observed. They included a 3rd chromosome carrying a deletion of the entire 3L arm, a 3rd chromosome carrying deletions in both arms, a 3rd chromosome carrying a pericentric inversion, an entire 2nd chromosome conjoined to a euchromatic arm of a major metacentric chromosome (an element of a translocation) (d), an entire 2nd chromosome conjoined with a small piece of heterochromatin (an element of a translocation), an acrocentric chromosome generated by a deletion of the entire arm of a major autosome (this deleted chromosome could be either 2L or 3R) (e), several small acrocentric chromosome probably derived from the major autosomes (f). We observed regular X chromosomes as well as an X chromosome with the long arm conjoined with autosomal euchromatic material (an element of a translocation) (d-f). Several types of dot chromosomes were observed, yet some of them appeared to be regular 4th chromosomes. All these chromosomes were found in various combinations in metaphases that often displayed chromosome breaks

Sex. In the 16 karyograms analyzed the average X:A ratio was 0.54 where we considered the amount of autosomal material and not the number of metacentric autosome elements (Table S1). The observed karyotype agrees with the sequencing data indicating that this line has a male karyotype and expression pattern.
